# Identifying volatile and non‐volatile organic compounds to discriminate cultivar, growth location, and stage of ripening in olive fruits and oils

**DOI:** 10.1002/jsfa.11805

**Published:** 2022-02-17

**Authors:** Maria Greco, Natasha Spadafora, Martin Shine, Ann Smith, Antonella Muto, Innocenzo Muzzalupo, Adriana Chiappetta, Leonardo Bruno, Carsten Müller, Hilary Rogers, M. Beatrice Bitonti

**Affiliations:** ^1^ Department of Biology, Ecology, and Earth Sciences University of Calabria Arcavacata di Rende Italy; ^2^ School of Biosciences, Cardiff University Cardiff UK; ^3^ Department of Chemical, Pharmaceutical and Agricultural Sciences University of Ferrara Ferrara Italy; ^4^ Centro di Ricerca Olivicoltura, Frutticoltura e Agrumicoltura, Consiglio per la Ricerca in agricoltura e l’analisi dell’economia agraria (CREA‐OFA) Rende Italy

**Keywords:** altitudinal effects, lipoxygenase pathway, *Olea europaea*, olive oil, volatile organic compounds

## Abstract

**BACKGROUND:**

There is increasing consumer demand for olive oil to be traceable. However, genotype, environmental factors, and stage of maturity, all affect the flavor and composition of both the olives and olive oil. Few studies have included all three variables. Key metabolites include lipids, phenolics, and a wide range of volatile organic compounds (VOCs), which provide the olives and oil with their characteristic flavor. Here we aim to identify markers that are able to discriminate between cultivars, that can identify growth location, and can discriminate stages of fruit maturity. ‘Nocellara messinese’ and ‘Carolea’ olive fruits were grown at three locations differing in altitude in Calabria, Italy, and harvested at three stages of maturity. Oil was analyzed from the two most mature stages.

**RESULTS:**

Nine and 20 characters discriminated all fruit and oil samples respectively, and relative abundance of two fatty acids distinguished all oils. Whole VOC profiles discriminated among the least mature olives, and oil VOC profiles discriminated location and cultivar at both stages. Three VOCs putatively identified as hexanal, methyl acetate, and 3‐hexen‐1‐ol differentiated all samples of oils from the most mature fruit stage.

**CONCLUSION:**

The results confirm that interactions of location, cultivar and fruit maturity stage are critical for the overall pattern of aroma compounds, and identify potential markers of commercial relevance. © 2022 The Authors. *Journal of The Science of Food and Agriculture* published by John Wiley & Sons Ltd on behalf of Society of Chemical Industry.

## INTRODUCTION

Olive (*Olea europaea* L subsp. *europaea* var. *europaea*) fruits (drupes) develop over a well‐defined pathway, the last stages of which involve accumulation of oils in the mesocarp followed by ripening. This involves softening, and a color change from green to green/purple (‘cherry’ stage) to purple/black.^1^, ^2^

Fatty acids (FAs) accumulate in the olive drupes throughout their development. In both olive drupes and oil, oleic acid (C18:1) can comprise up to 75–80% of total FAs, while the remaining minor percentage is represented by linoleic acid (C18:2), palmitic acid (C16:0), stearic acid (C18:0), and linolenic acid (C18:3).^3^ Both drupe and oil are also rich in important antioxidants including phenols, carotenoids, and tocopherols,^4^ which confer oxidative stability to olive products. Phenolics accumulate during olive fruit ripening reaching a maximum at the ‘cherry stage’.^5^ The phenolic composition of olive oil reflects that of the olive, although chemical changes to the phenolic profile also occur during oil extraction. Moreover, during drupe ripening a broad range of volatile organic compounds (VOCs), exhibiting ‘green’ or ‘grassy’ and ‘fresh’ notes are produced, acting as important aroma and flavor molecules, and affecting olive product quality perception.^1^

The key metabolic pathway responsible for the biosynthesis of VOCs in olive drupes is the lipoxygenase cascade associated with drupe softening.^1^ The first enzymes in this process are lipoxygenases (LOXs), which catalyze oxidation of polyunsaturated fatty acids (i.e. linoleic and linolenic acids) to form 13‐hydroperoxide derivatives of polyunsaturated fatty acids. The next step is their cleavage by hydroperoxide lyase (HPL) into C6 aldehydes, which then undergo reduction to C6 alcohols by alcohol dehydrogenases (ADHs). Alcohol acyl transferases (AATs) then transform these into the corresponding esters. Biogenesis of C5 and C6 volatile organic compounds is related to disruption of olive cells, which results in the release of lipid‐degrading enzymes when olive fruits are chopped or crushed (for example during the process of oil extraction). Volatile organic compound production is further enhanced by malaxation processes undertaken during oil production.

Clear quantitative and qualitative differences are observed among olive cultivars with respect to the time‐course of drupe development. These affect acyl composition, antioxidant metabolites, and VOC production.^6^, ^7^ Moreover, in addition to genotype, environmental factors and cultivation practices also strongly influence physiological processes and metabolic pathways underlying drupe development.^2^ These, in turn, affect the quality and sensory properties of olive products. In particular, the balance between saturated and unsaturated FAs is strongly influenced by temperature^2^ and it is known empirically that oil derived from plants growing at higher altitude produce better quality products. In addition, levels of antioxidants including phenolics and other active bio‐molecules are also modulated by altitude and other environmental variables, including cold damage (particularly freezing) during fruit ripening.^8^ Volatile organic compound production is also environmentally modulated, and VOC profiles for the same olive cultivar can differ across different growing regions.^9^

Currently, olive cultivation is increasing, spreading from the Mediterranean region where it originated, to non‐traditional production areas such as the southern hemisphere.^10^ At the same time, both European Directives and consumers demanding high food quality, require that the origin of olive products is clearly stated and is traceable with respect to both genotype and geographic cultivation area.^11^ Rapid and robust methods for discriminating origin and cultivar are therefore needed. Previous approaches to discriminate cultivars, olive maturity stage, or effects of environmental growth factors have included the analysis of fatty acid composition,^12^ phenolics, spectrophotometrically or by high‐performance liquid chromatography (HPLC)^13^, ^14^ and analysis of VOCs,^6^, ^9^, ^15^ with varying success in discrimination. Mid‐infrared spectroscopy^16^ and nuclear magnetic resonance (NMR)^17^ spectroscopy also show promise, combined with advanced statistical analyses such as principal component, partial least square discriminant, or multivariate analysis.

Here we assess VOC profiles and non‐VOC characteristic, to profile olives and olive oil from the same two cultivars at different stages of ripening and grown in contrasting geographical areas. The aims of our study were to determine whether we could use VOCs or combinations of VOCs and other characteristics to provide useful objective markers for tracing cultivation origin, and olive cultivars used for both olives and the oils derived from them, irrespective of the growth stage of the olive fruit. Such markers would be of potential use in the industry for tracing the source and cultivar of olives and olive products.

## MATERIALS AND METHODS

### Chemicals

All chemicals used to determine olive fruit and oil volatile and non‐volatile characteristics were of analytical or HPLC grade, purchased from Sigma‐Aldrich (Gillingham, Dorset, UK or Milan, Italy), VWR (Lutterworth, Leicestershire or Milan, Italy) or Fisher (Loughborough, UK or Rodano (MI), Italy). Standards for phenolics, tocopherol and fatty acid were purchased from Sigma‐Aldrich.

### Source of olive materials and collection of volatile organic compounds

Olive fruit and oil samples were from two olive cultivars. Oil from cv. Carolea has a more delicate flavor described as having notes of grass, almond and artichoke with bitter notes, and medium in spicy taste. Cv. Nocellara messinese oil has a stronger flavor with almond and tomato notes, medium bitterness, and spicy flavor.^18^ Clonal populations of olive trees were grown at three different locations near Cosenza, in Calabria, Italy: Mirto Crosia (39° 36' 9.11'' N, 16° 46' 3.71'' E), Rende (39° 19' 54.39'' N,16° 11' 2.05'' E), and Mongrassano (39° 31' 38.4'' N, 16° 6' 56.25'' E; for map see supporting information, Fig. S1A). Olive trees at Mirto Crosia were grown at 8 m above sea level (a.s.l.) at a regional agricultural development station (Agenzia Regionale per lo Sviluppo dell’Agricoltura Calabrese, ARSAC). In Rende (225 m a.s.l.) olive trees were grown at the Research Centre for Olive, Fruit and Citrus crops (CREA‐OFA). At Mongrassano (540 m a.s.l.) the olives were grown on a farm. Trees of both cultivars from Mirto Crosia and Rende were 20–25 years old, whereas cv. Nocellara messinese and cv. Carolea trees from Mongrassano were 15 and 60 years old respectively, but all trees were at peak olive productivity. Olive trees were not irrigated and were pruned according to normal agronomic practice: removing suckers and pruning the crown as necessary. All trees used in the study were genotyped to verify the cultivar using microsatellites (data not shown) as described previously.^19^ The temperature was recorded throughout the harvest period starting 1 week before the first harvest date (supporting information, Fig. S1B). The temperature at Mirto Crosia and Mongrassano was relatively similar, whereas the temperature at Rende was on average 2 °C lower throughout this period. Olives were collected based on a visual assessment of their maturity stage to represent three different stages of fruit maturity (1, 2, 4, and)^20^ in the 2013/2014 season. Stage 1 olives were 100% green and were picked approximately 150 days after flowering (DAF), stage 2 olives were 20% yellow (picked at ~160 DAF), stage 4 olives were 50–60% purple (picked at ~180 DAF). To verify that the stages of maturity were indeed equivalent for olives collected at the three growing sites, olive size and weight was measured for both cultivars at each stage of fruit development and each growth site, and no significant differences across sites were found. For each population of trees and at each sampling, at least 20 kg of olive fruit were hand‐picked from at least ten individual trees. To minimize effects related to asynchronous maturation of fruits within the same tree, fruit were only collected from external parts of the tree canopy. Only fruits not showing recognizable signs of damage (e.g. caused by infection with *Bactrocera oleae*) were collected, and were divided into three biological replicates. After harvesting, 10–15 kg of drupes were immediately processed for oil extraction using an Oliomio milling machine (Toscana Enologica Mori, Tavarnelle Val di Pesa, Florence, Italy, a small‐scale industrial machine suitable for processing 25–75 kg of olives).^21^ The paste obtained was mixed at room temperature for 30 min, and the oil extracted was centrifuged (1300×*g* for 3 min) to eliminate residues of water, air, proteins, enzymes, pectins, mucilage, etc. The clarifying effect obtained is comparable to filtration. The supernatant (clarified virgin olive oil) was transferred into dark glass bottles and stored at 4 °C prior to analysis. Oil was only extracted from fruit at stages 2 and 4 of maturity.

### Chemical composition and maturity index of olive fruits

Chlorophylls were extracted with acetone from 100 mg of freeze‐dried olive drupe pericarps ground in liquid nitrogen, with three biological replicates.^22^ Absorbance at 646.8 and 663.2 nm was used to calculate the content of chlorophyll a and b.^23^

Total phenolics were extracted from 200 mg of ground olives (with three biological replicates). Three extractions with 15 mL of methanol/acetone (1:1), saturated with sodium disulfite for 20 min, were followed by centrifugation at 5000×*g* at 4 °C for 20 min. The supernatants were combined, evaporated to dryness under vacuum at 45 °C, and the residue dissolved in 8 mL water. The aqueous solution was extracted once with 8 mL hexane, and phenolics were extracted with 8 mL ether/ethyl acetate (1:1) six times. The combined ether/ethyl acetate extracts were dried with sodium sulphate and evaporated to dryness under vacuum at 30 °C. The residue was resuspended in 5 mL methanol. Spectrophotometric determination of phenolics was as described for olives except 0.02 mL of the methanolic extract was used.

Ripeness (Jaén index) was determined according to the guidelines of the Spanish National Institute of Agronomic Research based on a subjective evaluation of olive skin and pulp colors from a randomly selected sample of 100 fruits grouped as three biological replicates.^20^ Lipid content was determined gravimetrically after Soxhlet extraction with hexane for 6 h.^21^ Results calculated from triplicate data are expressed as means ± standard deviations.

Sugars in olive drupes were extracted from 16 g olive paste. They were extracted once with 150 mL of water with addition of 5 mL of each Carrez solution I and II and sonication at room temperature for 15 min. After filtration and recovery of the pulp, the procedure was repeated with another 150 mL of water. Equal volumes of both extracts were combined and stored at 2–8 °C for analysis. Sugars were resolved isocratically over a 15 cm × 2.1 mm, 5 μm Chromegabond carbohydrate column (ES industries, West Berlin, NJ, USA) with 8/2 acetonitrile/54 μM Cs in water as mobile phase. Sugars were detected by ESI‐MS–MS using the [M+ Cs]^+^ M + Cs^+^ transition at an ion spray voltage of 5500 V; source temperature of 100 °C, curtain gas pressure of 10 psi, collision energy of 25 V, declustering potential of 100 V, and entering potential of 10 V. Retention times and concentrations were determined against standard solutions and dilutions of fructose (0.552 mg/g), glucose (0.274 mg/g), galactose (0.176 mg/g), mannitol (0.128 mg/g), and sucrose (0.194 mg/g).^24^ Three biological replicates were analyzed.

### Chemical composition and quality characteristics of olive oil

Free acidity, peroxide value, and UV light absorption (K_232_, K_270_, *Δ*K) of oil were determined following EC Regulation 2568/91^25^ and subsequent amendments and additions (EEC Regulation no. 2568/91). The mean of the data was calculated from three biological repeats.

The fatty acid composition of oil was determined as fatty acid methyl esters (FAMEs) (EEC Regulation no. 2568/91). Briefly, 0.15 g of oil was dissolved in 1 mL of hexane; 0.1 mL of a methanolic solution of KOH (1 mol/L) was added, and the mixture was shaken vigorously for 5 min and allowed to separate. Fatty acid methyl esters were recovered in the supernatant, of which 0.25 mL were mixed with 1.5 mL hexane. Finally, 1 μL of the resulting solution was analyzed with a gas chromatgraph with flame ionization detector (GC‐FID). Fatty acid methyl esters were separated over 60 m, 0.25 mm ID, 0.2 μm SP‐2340 (Supelco) using an Agilent 6890N gas GC‐FID (at 260 °C) (Agilent Inc., Santa Clara, CA, USA) and the following temperature program: start temperature 110 °C for 5 min, 3 °C min^−1^ to 150 °C, 16.33 min hold at 150 °C, 4 °C min^−1^ to 230 °C and final hold for 27 min at 230 °C. Peaks were identified by directly injecting a solution into the GC‐FID containing a mixture of standards and comparing the retention times of each compound to known standards. The standards were: methyl myristate, >98%, CAS number 124‐10‐7; methyl palmitate, >99%, CAS number 112‐39‐0; methyl heptadecanoate, >99%, CAS number 1731‐92‐6; methyl stearate, >99%, CAS Number 112‐61‐8; methyl *γ*‐linolenate, >99%, CAS Number16326‐32‐2; methyl oleate, >99%, CAS number 111‐62‐9; methyl linoleate, >98%, CAS number 112‐63‐0 (Sigma‐Aldrich, Milano, Italy). Relative retention times were also calculated as the ratio between the retention time of each compound and that of the internal standard. The results are expressed as percentages of chromatographic areas^12^ from a mean of three biological replicates.

For the analysis of tococopherol, 6 g oil was dissolved in hexane and made up to 10 mL. The solution was filtered with a Polytetrafluoroethylene (PTFE) filter (0.2 μm, 25 mm, Whatman, Kent, UK), and 20 μL were analyzed by HPLC (Agilent 1100, Milano, Italy) over a 25 cm, 4.6 mm, 5 μm Zorbax NH2 column (Agilent, Milano, Italy) in isocratic mode with 2 mL min^−1^ hexane: ethyl acetate (80:20, V:V). Tocopherols were detected by fluorescence spectrophotometry at 295 and 325 nm. The results are expressed as the sum in mg of *α*, *β*, *γ* and *Δ* tocopherol per kg of oil.^22^ The mean of the data was calculated from three biological replicates.

Total phenolic compounds were determined spectrophotometrically after solid‐phase extraction on LiChrolut RP18 cartridges (40–63 mm, 1000 mg/6 mL PP‐tubes, Merck, KGaA, Germany). SPE cartridges were conditioned with 2 × 6 mL of methanol followed by 2 × 6 mL of *n*‐hexane. Olive oil (1 g) was dissolved in 6 mL of *n*‐hexane and applied to the cartridge. Samples were washed with 3 × 6 mL of *n*‐hexane and eluted with 3 × 6 mL of methanol. The methanolic solution was dried in a rotary evaporator (R‐300, Buchi, Flawil, Switzerland) at 35 °C, the dry residue dissolved in 1 mL of methanol, filtered and stored at −20 °C^26^. For spectrophotometry, 0.2 mL of the methanol solution was diluted to 2.5 mL with water and 0.25 mL Folin–Ciocalteu reagent was added. After 3 min, 0.5 mL Na_2_CO_3_ solution (35%, w/v) was added to the reaction mixture, mixed, and diluted with water to 5 mL, and left to react for 2 h. Total phenolics were determined from absorbance at 725 nm against a standard curve of caffeic acid (Sigma‐Aldrich) from 1 to 5 mgL^−1^ using a JASCO V‐530 spectrophotometer (JASCO, Cremella (Co), Italy). The results are reported as mean values of three biological replicates.

### Collection and analysis of volatile organic compounds (VOCs)

Volatile organic compounds (three biological replicates) were collected and analyzed essentially as previously described.^27^ An Easy‐VOC pump system was used to sample VOCs from the headspace of 100 g of chopped olive fruit or 50 mL of olive oil, and incubated at 25 °C in nalophan bags for 2 hours to equilibrate. The headspace (300 mL) was passed through SafeLok tubes (Markes International Ltd, Bridgend, UK) packed with Tenax TA and SulfiCarb sorbents. Samples were also collected from empty bags as controls. Retention index standards (1 μL of C8‐C20 alkanes; Sigma Aldrich), and internal standards (benzene 1,4‐difluoro, chlorobenzene‐d5 and *p*‐bromofluorobenzene, Restek) were loaded directly into TD tubes. Tubes were desorbed on a TD100 thermodesorption system (Markes International Ltd.) for 5 min at 100 °C, then 5 min at 280 °C, with a trap flow of 40 mL min^−1^. For trap desorption and transfer into the GC (7890A; Agilent Technologies), 20 °C s^−1^ to 300 °C s−1, and a split flow of 5 mL min^−1^ were used. To separate the VOCs, a 60 m, 0.32 mm I.D., 0.5 μm Rx5ms column (Restek, Bellefonte PA, USA) with 2 mL min^−1^ helium as carrier gas under constant flow conditions was used. Initial temperature was set to 40 °C for 2 min, with 5 °C min^−1^ to 240 °C, and a final hold 5 min. A time‐of‐flight mass spectrometer (BenchTOF‐dx, Markes International Ltd) was used to record mass spectra from m/z 30–350.

Gas chomatography–mass spectrometry data were inspected using MSD ChemStation software (E.02.01.1177, Agilent Technologies, Inc.) and were then deconvoluted and integrated with AMDIS (National Institute of Standards and Technology (NIST) Standard Reference Data Program) using a custom retention‐indexed mass spectral library. Mass spectrometry spectra from deconvolution were searched against the NIST 2011 library^28^ (version 2.0). Volatile organic compounds scoring >80% in forward and backward fit and a retention index (RI) match of ±15 were included into the custom mass spectral library as putatively identified VOCs. Volatile organic compounds scoring >80% in forward and backward fit and no RI match were included as chemical class, e.g. alkane, alkanol. Recurrent components that did not show either the required mass spectral fit or RI match were added as ‘unknown’. Peak lists from integration with AMDIS were aligned using the pivot function in Excel. Compounds were removed if they did not appear in multiple replicates for any condition, if they were a known contaminant, or if the average integrated signal (IS) was less than 10× higher than that of the IS of the controls for that compound. The IS measurements were converted into percentage of the grand total of the VOC area recorded for that sample to derive the relative abundance per sample for each VOC and then they were square‐root transformed to reduce the weight of large components.

### Statistical analysis

Differences in VOC and other characteristics were analyzed using permutational multivariate analysis of variance (PerMANOVA), and canonical analysis of principal coordinates (CAP) using RStudio software Version 1.1.383 (R version 3.5.2) as detailed previously.^29^ This used the ‘adonis’ function in the package ‘vegan’ and ‘CAP‐discrim’ function in the package ‘BiodiversityR’. Permutational multivariate analysis of variance^30^ is a non‐parametric multivariate test that enables testing of significant differences of groups of characteristics for factors (here, cultivar, growth location, maturity stage) and their interaction. Canonical analysis of principal coordinates is based on an analysis of principal ordinates (POs) and considers individual factors separately. It carries out a linear discriminant analysis of the POs, to test the hypothesis that the data are able to discriminate between the samples and assigns a percentage correct classification.^31^ Linear discriminant plots from the CAP analysis were produced for cultivar, growth location, stage of maturity and sample, and a 95% confidence interval was fitted. Heat maps were produced in R, and Random Forest (RF) in Metaboanalyst was used as an unsupervised machine‐learning algorithm.^32^ Random Forest creates multiple decision trees to categorize a training set of the data and then tests the accuracy of the derived categories across the remaining data. It then ranks the importance of the characteristics in assigning the categories generating a ‘mean decrease accuracy’ table.

Differences amongst non‐VOC characters were also analyzed using ANOVA or Kruskal–Wallis tests followed by an Least Significant Difference (LSD) or Dunn’s test with Benjamini–Hochberg correction, chosen depending on results from a Fligner–Killeen test for homogeneity of variances and a Shapiro–Wilk normality test.

## RESULTS

### Differences in phytochemical and physiological characteristics across olive samples

**Figure 1:**
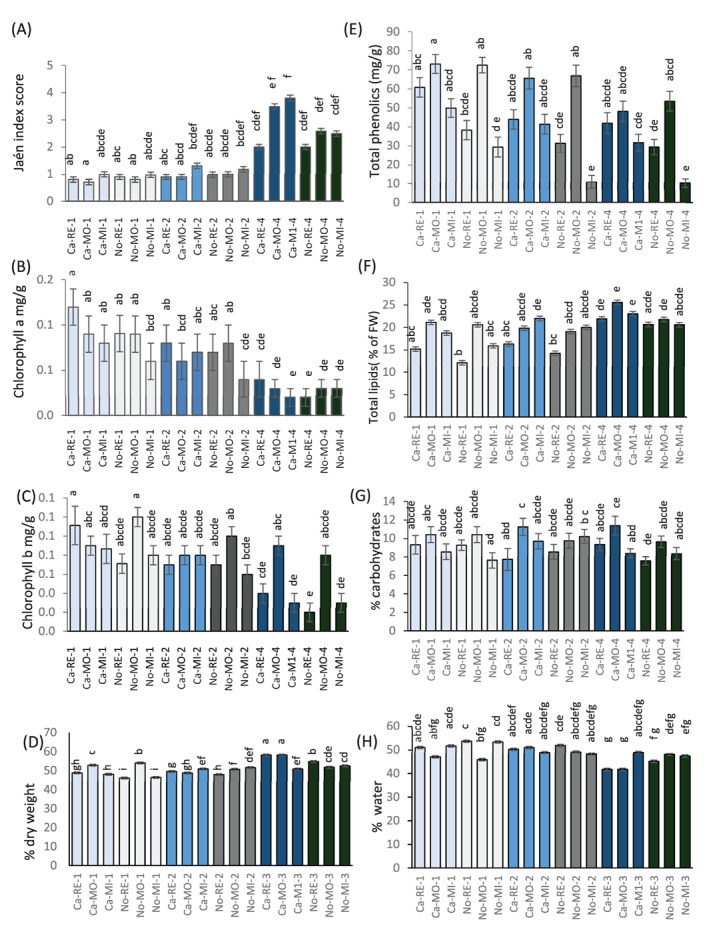
Physiological and phytochemical characteristics of olive fruit at three developmental stages (1, 2, and 4) from olives of two cultivars (Ca, ‘Carolea’; No, ‘Nocellara messinese’) grown in three different locations (RE, Rende; MO, Mongrassano, MI, Mirto). Jaén index of fruit maturity (A), chlorophyll a (B), chlorophyll b (C), percentage dry weight (D), total phenolics (E), total lipids (F) carbohydrate content (G) and percentage water content (H). *n* = 3, ± SD, letters indicate significant differences amongst developmental stages (*P* < 0.05) based on one‐way ANOVA or the Kruskal–Wallis test followed by an Least Significant Difference (LSD) or Dunn’s test with a Benjamini–Hochberg correction.

Chlorophyll content decreased significantly with ripening, while the Jaen index increased, but neither showed significant differences across cultivar (‘Carolea’ or ‘Nocellara’) or growth location (Mirto Crosia, Rende or Mongrassano) confirming that olives were indeed harvested at equivalent stages of maturity. Overall, stage 1 (100% green) and stage 2 (20% yellow epicarp) were much less distinct from each other than the ripest stage (Fig. 1(A)–(C) – the raw data are in the supporting information, in Table S1A). At the ripest stage (stage 4, 50–60% purple), ‘Carolea’ olives grown at Mongrassano contained significantly more chlorophyll b than those grown at Mirto Crosia (*P* < 0.05; Fig. 1(C)).

Total phenolics were highly abundant, reaching a maximal level of over 70 mg/g. Abundance varied with growth location: olives grown at Mongrassano generally contained more phenolic compounds although differences were more marked in the first two stages of ripening and were not always statistically significant (Fig. 1(E)). Phenolic compounds mostly decreased with ripening but with no consistent differences linked to cultivar or growth location. Total lipids were the most abundant group of metabolites; they tended to increase with ripening but showed no consistent differences related to cultivar or location. Olives of both cultivars grown at Mongrassano often had a higher lipid content than olives from the other locations, although differences were not always statistically significant (Fig. 1(F)). There were very few significant differences overall in carbohydrate content related to cultivar or growth stage (Fig. 1(G)), although percentage water content showed few significant differences across cultivar or growth location (Fig. 1(H)), and in the youngest stage dry weight was higher in olives grown in Mongrassano, whereas at later stages there was no consistent difference (Fig. 1(D)).

### Olive fruit characteristics discriminate by cultivar and location at three stages of maturity

To assess whether the eight phytochemical and physiological characteristics (Fig. 1) plus total chlorophyll (raw data in supporting information, Table S1A) could discriminate amongst the olive samples, permutational multivariate analysis of variance (PerMANOVA) was applied across all three stages of olive maturity. These nine olive fruit characteristics were able to discriminate amongst samples (*P* < 0.001; *R* ^2^ = 0.91). Differences were significant for location (*P* < 0.001; *R* ^2^ = 0.21) and cultivar *P* < 0.001; *R* ^2^ = 0.042), but there was also interaction between location and cultivar, and location and stage (*P* < 0.01), although no interaction between cultivar and stage (supporting information, Table S1C).

**Figure 2:**
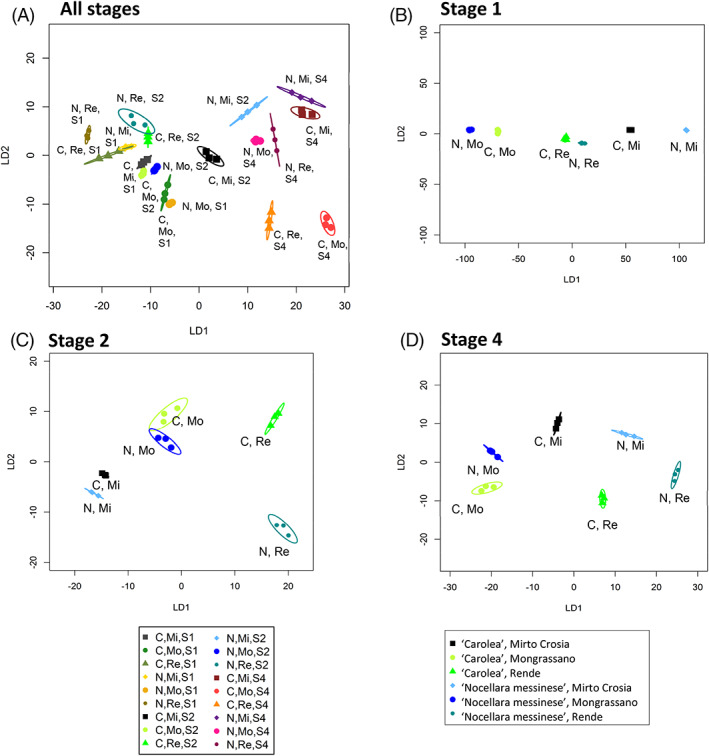
Linear discriminant plots from canonical analysis of principal coordinates based on olive fruit phytochemical and physiological characteristics. Canonical analysis of principal coordinates models were produced for samples from different locations (Mirto Crosia (Mi), Mongrassano (Mo), Rende (Re)) and cultivars (‘Nocellara messinese’ (N), ‘Carolea’ (C)) for olives of (A) all stages, (B) stage 1 (S1), (C) stage 2 (S2), and (D) stage 4 (S4). Ellipses represent the 95% confidence interval (SD). Percentage of correct classifications, (A) 93% (*P* = 1), (B) 100% (*P* < 0.0001), (C) 94.4% (*P* = 0.58), (D) 100% (*P* < 0.001).

Linear discriminant plots were produced from canonical analysis of principal coordinates (CAP) (Fig. 2). When all the stages were considered together (Fig. 2(A)), 14 out of the 18 samples showed unique patterns but there were two pairs of samples that could not be discriminated. These were stage 2 olives from the two cultivars both grown at Rende, and stage 1 ‘Nocellara messinese’ olives grown at Mirto Crosia compared with ‘Carolea’ olives grown at Rende. When each stage of olive maturity was considered separately (Fig. 2(B)–(D)), all samples were discriminated by location and by cultivar apart from stage 2 olives grown at Mongrassano, where the two cultivars could not be discriminated. At all three stages of development, the nine characteristics discriminated growth location when cultivars were combined. In contrast, cultivars could only be discriminated at fruit maturity stages 2 and 4 when locations were combined (supporting information, Fig. S2).

### Three characteristics were most discriminatory amongst olive samples

**Figure 3:**
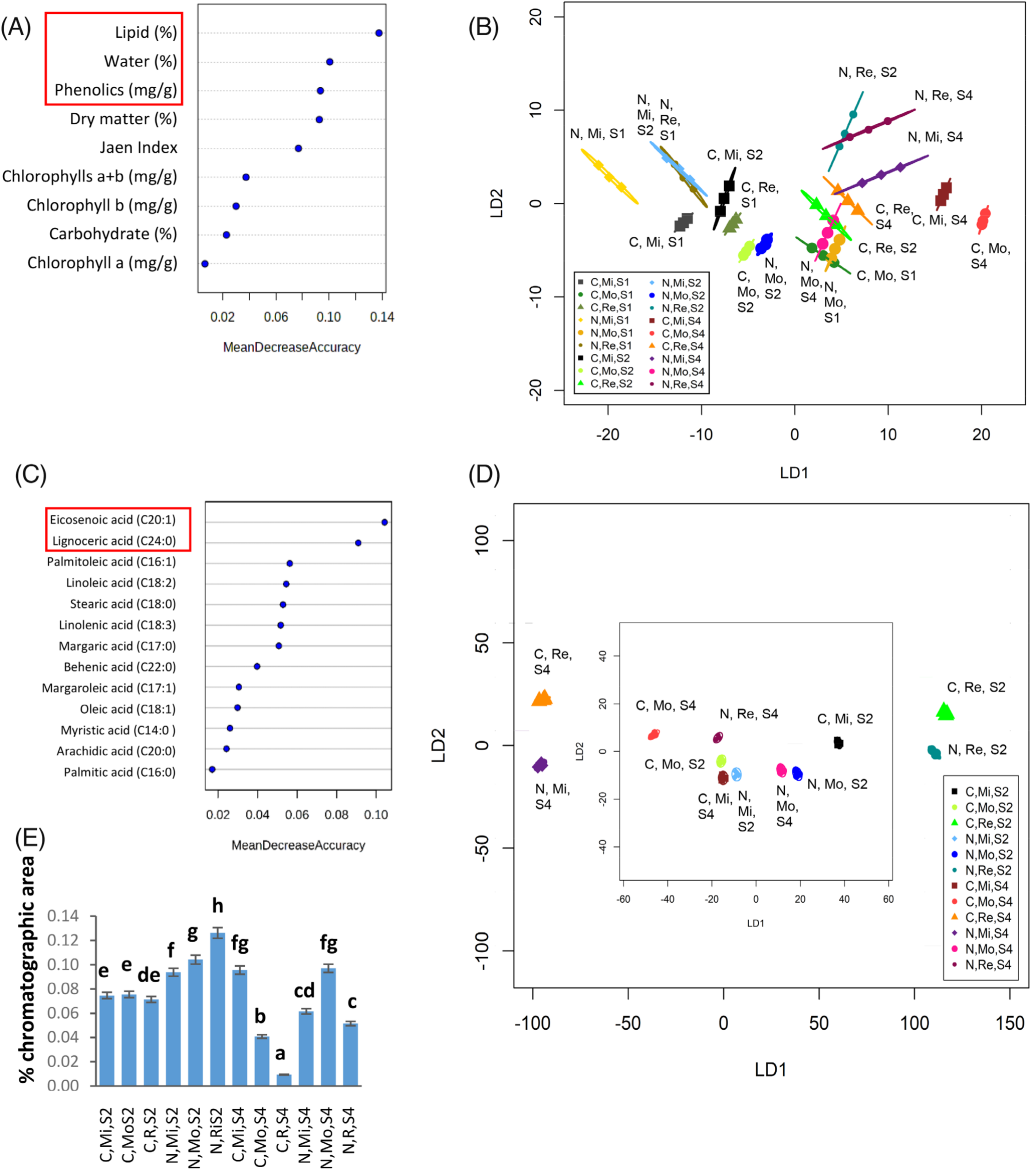
Most discriminatory characteristics for olives, and fatty acid composition of all oil samples. Random Forest analysis of biochemical parameters in olives (A) and fatty acids in oils (C) to identify key discriminators. Canonical analysis of principal coordinates (CAP) based on top discriminators (B) lipid, water and phenolic (%) in olives of all three stages (1, 2, 4, and) and (D) eicosenoic acid (C20:1) and eignoceric acid (C24:0) (%) in both oil stages (2 and 4). The first two linear discriminants were used, and each ellipse represents the 95% confidence interval (SD). Mirto Crosia (Mi), Mongrassano (Mo), Rende (Re)) and cultivars (‘Nocellara messinese’ (N), ‘Carolea’ (C)) for olives of (A) all stages (B) stage 1 (S1), (C) stage 2 (S2) and (D) stage 4 (S4). Percentage of correct classification for CAP was (B) 93% and (D) 100%, *P* < 0.01, *n* = 3. (E) Percentage of chromatographic area of eicosenoic acid (C20:1), lower case letters indicate significant differences (*P* < 0.05) based on a one‐way ANOVA followed by a Tukey test.

We used RF analysis to identify the characteristics that most influenced discrimination across the olive samples. Three of the nine olive characteristics – lipids, water content, and total phenolics – were identified as most important in their discriminatory power amongst olive samples, based on the RF mean decrease accuracy (Fig. 3(A)). Based on the data from these three characteristics, discrimination was retained using PerMANOVA (*P* < 0.001; *R* ^2^ = 0.964), and a linear discriminant plot based on CAP analysis (Fig. 3(B)) separated many of the samples, although only eight of the 18 samples were fully discriminated from each other. At each stage of development, the two cultivars were discriminated when grown at Mirto or Rende. However, stage 1 olives from the two cultivars grown at Mongrassano could not be discriminated from each other using only these three characteristics. When each cultivar was considered separately, olives could be discriminated on the basis of location within each growth stage.

### Olive VOC profiles are affected by cultivar, stage of fruit maturity, and location of growth

Twenty‐six different VOC compounds were identified tentatively in the aroma of the olive samples based on matches to the NIST database, across all locations and cultivars (supporting information, Table S2). The profile comprised most aldehydes (five) and alcohols (five) followed by acetate esters and alkanes (three of each), two each of non‐acetate esters, ketones and sulfur compounds, and one amine, aromatic compound, ether and furan derivative. The three VOCs with highest mean relative abundance across all samples were putatively identified as hexanal (C40), (Z)‐3‐hexen‐1‐ol (C21) and dimethyl sulfide (C35). Whole VOC profiles were significantly different between the three ripening stages (PerMANOVA, *P* < 0.001, *R* ^2^ = 0.327), and across the three locations (*P* < 0.01, *R* ^2^ = 0.066), although there was interaction between location and stage (supporting information, Table S1C; raw data in supporting information, Table S3A). Within individual olive stages, VOCs did not discriminate by location or cultivar.

**Figure 4:**
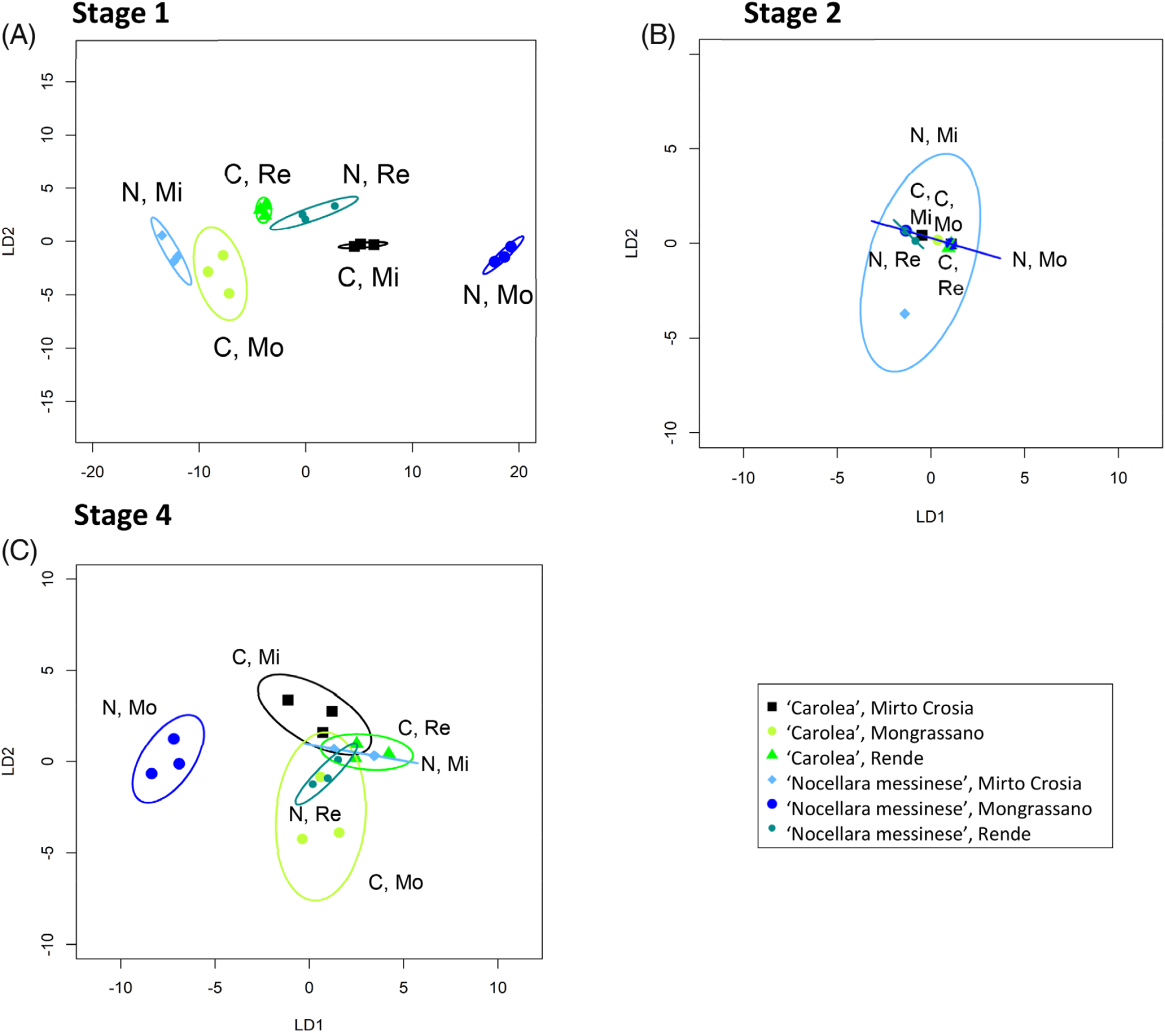
Linear discriminant plots from canonical analysis of principal coordinates based on VOCs in olives. Canonical analysis of principal coordinates (CAP) models were produced for samples from different locations (Mirto Crosia (Mi), Mongrassano (Mo), Rende (Re)) and cultivars (‘Nocellara messinese’ (N), ‘Carolea’ (C) of olives of stage 1 (A), stage 2 (B) and stage 4 (C). Ellipses represent the 95% confidence interval (SD). Percentage of correct classifications, (A) 94.4% (*P* = 1), (B) 39% (*P* < 0.99), (C) 56% (*P* = 0.75).

Linear discrimination plots based on CAP did not separate whole VOC profiles amongst stages of ripening (supporting information, Fig. S3A), by cultivar (supporting information, Fig. S3C, E, G) or growth location (supporting information, Fig. S3B, D, F) at any of the three stages. However, at stage 1 there was a correct classification of 94.4%, and clear separation across all samples (Fig. 4). At stage 2 and stage 4 correct classification fell to 39% and 56% respectively. At stage 2, VOC profiles only discriminated olives from the two cultivars grown at Rende. Only ‘Carolea’ olives could be discriminated across the three growth locations, while ‘Nocellara messinese’ olive profiles were not distinct. At stage 4, olive VOC profiles could only discriminate between cultivars when they were grown at Mongrasano. The VOCs could not discriminate ‘Carolea’ olives by growth location, but ‘Nocellara messinese’ olives grown at Mongrassano were discriminated from those grown at the other two locations.

### Differences in non‐VOC characteristics across olive oil samples

Twenty different non‐VOC oil characteristics were assessed across samples. Oil extracted from olives grown at Mirto Crosia tended to have higher acidity than oil extracted from those grown at the other two sites (supporting information, Fig. S4A), although differences were not always significant *(P* < 0.05) and there were no consistent differences in peroxide levels (supporting information, Fig. S4B; raw data in supporting information, Table S1B). Tocopherol content was lower in oil extracted from olives at stage 4 compared to stage 2 (supporting information, Fig. S4C), but there were no consistent differences between cultivars or amongst locations. However, total phenolics were higher in oil made from olives of both cultivars grown at Mongrassano (supporting information, Fig. S4D).

The fatty acid composition of the oil from individual samples differed significantly amongst growth locations, cultivars, and growth stage of the olives (PerMANOVA, supporting information, Table S4B), although only C14:0 (myristic acid) showed an interaction between the different variables. Oil extracted from stage 2 olives showed more differences in fatty acid composition between cultivars at the same location, and there were subtle, but not consistent, differences in the abundance of individual fatty acids related to growth location of the olives within the two cultivars (supporting information, Fig. S5; statistical analysis in supporting information, Table S4A).

**Figure 5:**
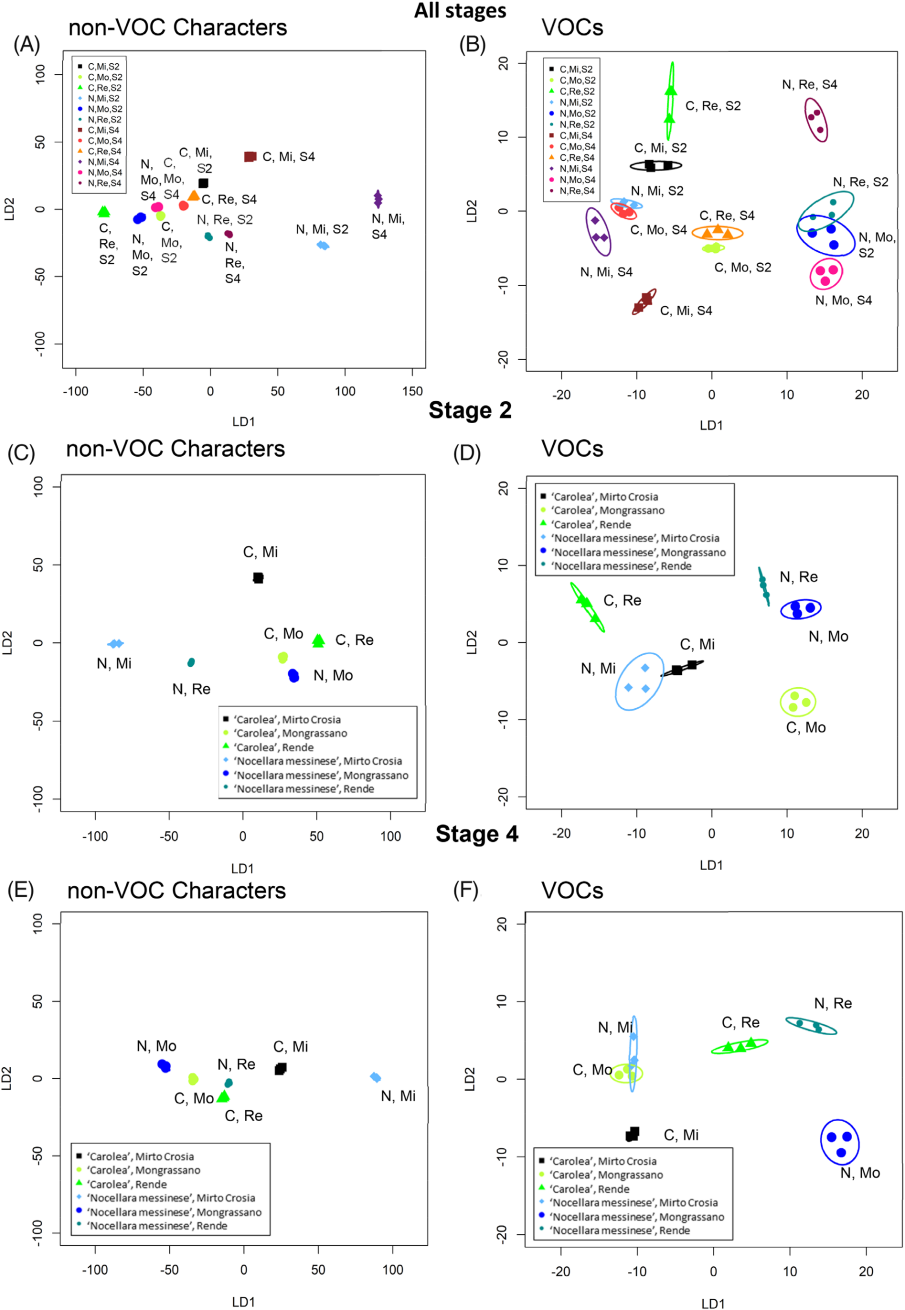
Linear discriminant plots from canonical analysis of principal coordinates based on VOCs and non‐VOC characteristics of olive oil. Canonical analysis of principal coordinates (CAP) models were produced for samples from different locations (Mirto Crosia (Mi), Mongrassano (Mo), Rende (Re)) and cultivars (‘Nocellara messinese’ (N), ‘Carolea’ (C)) for olive oil produced from both stages (S2 and S4) (A,B), stage 2 (C,D) and stage 4 (E,F) olives, from their non‐VOC characteristics (A,C,E) and VOC profiles (B,D,F). The first two linear discriminants were used, and each ellipse represents the 95% confidence interval (SD). Percentage of correct classifications: (A) 100% (*P* < 0.01), (B) 97%, (*P* = 1) (C) 100% (*P* < 0.01), (D) 100% (*P* = 0.01) (E) 100% (*P* < 0.001), (F) 100% (*P* = 0.01).

When all non‐VOC characteristics were taken together, samples were clearly differentiated by CAPdiscrim when oils from differing olive maturity were considered separately (Fig. 5(A), (C), (E)), and by PerMANOVA when all stages were considered together (*P* < 0.001; *R* ^2^ = 0.96) and by location (*P* < 0.001; *R* ^2^ = 0.385), cultivar (*P* < 0.001; *R* ^2^ = 0.15), and stage of fruit maturity (*P* < 0.001; *R* ^2^ = 0.161). However, PerMANOVA also revealed interactions amongst growth location, cultivar and stage (supporting information, Table S1C). Oils were clearly separable using non‐VOC characteristics both by location (supporting information, Fig. S6A) and olive growth stage (supporting information, Fig. S6C) but not by cultivar (supporting information, Fig. S6B) when other variables were pooled. However, if only oil extracted from stage 2 olives was included, then cultivars were also distinct (supporting information, Fig. S6E).

### Relative abundance of two fatty acids discriminates all oil samples

The two most discriminatory fatty acids based on RF analysis were eicosenoic acid (C20:1), and lignoceric acid (C24:0) (Fig. 3(C)). A linear discriminant plot across all the oil samples showed complete discrimination with a correct classification of 100%, *P* < 0.01 (Fig. 3(D)). Indeed relative abundance of eicosenoic acid (C20:1) alone was sufficient to discriminate between oils extracted from the two cultivars at each of the three locations at both stages of olive ripeness. It could also discriminate the three locations of growth from stage 2 ‘Nocellara messinese’ oil, and ‘Carolea’ stage 4 oil (Fig. 3(E)).

### Olive oil VOC profiles provide better discrimination than olive fruit VOC profiles

A total of 41 different VOCs were detected across all oil samples; 22 were unique to the oil, while 19 were present in both olives and the oil (supporting information, Table S2). Aldehydes formed the largest family of VOCs (eight). Remaining VOCs were alcohols and alkenes (six of each), alkanes and ketones (five of each), esters (three), acetate esters, terpenes and sulfur containing VOCs (2 of each), one furan and one aromatic VOC. The three VOCs with highest mean relative abundance across all samples were putatively identified as ethyl acetate (C36), 3‐hexenal (C22), and hexanal (C40).

There were significant differences between the VOC profiles of the oil from the three locations, the two cultivars, and the two stages (PerMANOVA, *P* < 0.001, *R* ^2^ = 0.191, 0.109, and 0.050 respectively). However, there were significant interactions amongst cultivar, location and stage (supporting information, Table S1C; raw data in supporting information, Table S3B). Analysis using CAPdiscrim showed that VOCs did not discriminate oil between cultivars, olive growth location or growth stage when all the samples were considered together (supporting information, Fig. S7A–C). However, when the oils from the two stages were considered separately, VOCs from each stage of olive discriminated both growth location and cultivar (supporting information, Fig. S7D–G).

Volatile organic compound profiles were able to discriminate most samples when oils of both olive stages were considered together (Fig. 5(b)) although profiles from ‘Nocellara messinese’ stage 2 olives grown at Rende and Mongrassano were not distinct, nor were ‘Nocellara messinese’ stage 2 olives grown Mirto Crosia and stage 4 ‘Carolea’ olives grown at Mongrassano. When stages were considered separately, VOCs discriminated most samples (Fig. 5(D), (F)), but not between the two cultivars grown at Mirto Crosia from stage 2 olives or oil extracted from ‘Nocellara’ olives grown at ‘Mirto Crosia’ and ‘Carolea’ olives grown at Mongrassano at stage 4.

### Three VOCs discriminate across all samples of oils extracted from stage 4 olive fruits

**Figure 6:**
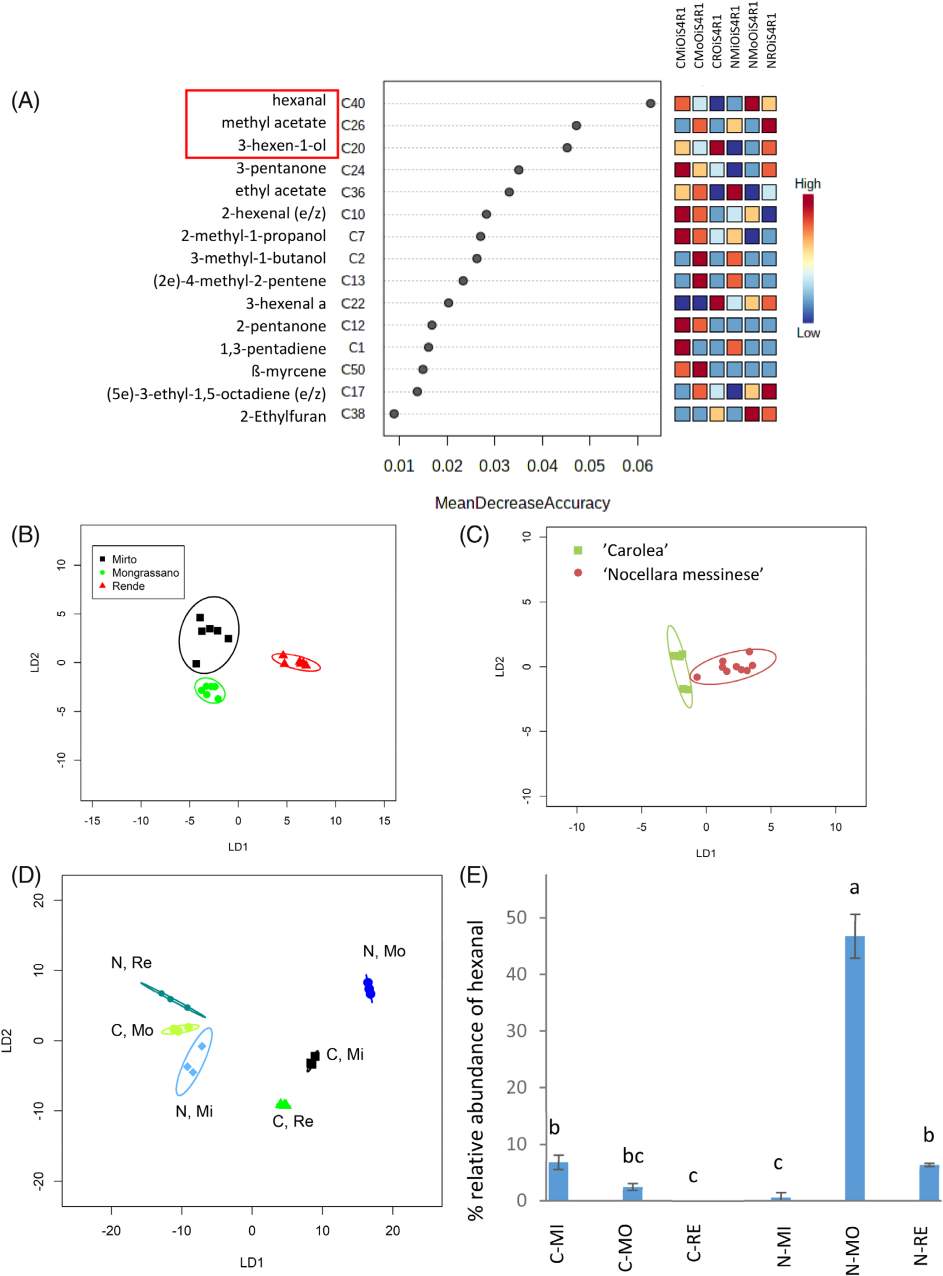
Analysis of VOCs from stage 4 olive oil (A) Random Forest identifies the 15 most discriminatory VOCs across all samples, CAP models were produced for samples from different locations (Mirto Crosia (Mi), Mongrassano (Mo), Rende (Re)) using relative abundance of the three most discriminatory VOCs: hexanal (C40), methyl acetate (C26) and 3‐hexen‐1‐ol (C20) according to (B) location (C) cultivars (‘Nocellara messinese’ (N), ‘Carolea’ (C)) and cultivar × location (D). Relative abundance of hexanal (C40) across all stage 4 oil samples (E), lower case letters indicate significant differences (*P* < 0.05) based on a one‐way ANOVA followed by a Tukey test. The first two linear discriminants were used for the CAP analysis and each ellipse represents the 95% confidence interval (SD). Percentage of correct classification where *n* = 6, 9, and 3 respectively, was (B) 94%, (*P* = 0.67) (C) 94%, (*P* = 0.01) and (D) 89% (*P* = 1).

Three VOCs were identified as the most important discriminators in Stage 2 olive oil using RF (supporting information, Fig. S8A). These were putatively identified as 2‐methyl‐1‐propanol (C7), ethyl acetate (C36), and methyl acetate (C26). However, although their relative abundance was significantly different across olive growth location, cultivar, and sample in PerMANOVA (*P* < 0.001, 0.05, 0.001; *R* ^2^ = 0.625, 0.028, and 0.974 respectively), there was significant interaction between location and cultivar (*P* < 0.001). Furthermore, they were unable to discriminate fully by location (supporting information, Fig. S8B) or by sample (Fig. S8D) in CAP linear discriminant plots. In contrast, the relative abundance of the top three VOC discriminators, putatively identified as hexanal (C40), methyl acetate (C26) and 3‐hexen‐1‐ol (C20), in oils extracted from stage 4 olive oil (Fig. 6(A)) discriminated the oil by olive growth location, cultivar, and across all samples both by PerMANOVA *P* < 0.001, *R* ^2^ = 0.310, 0.153 and 0.970 respectively), and in linear discriminant plots following CAP (Fig. 6(B)–(D)). However PerMANOVA revealed significant interaction between location and cultivar (*P* < 0.001). The relative abundance of the putative hexanal (C40) alone discriminated oil from ‘Nocellara messinese’ olives grown at the three locations and between cultivars when grown at each location, but oil from ‘Carolea’ olives grown at Mongrassano was not distinct from that derived from olives grown at Mirto Crosia or Rende (Fig. 6(e)).

## DISCUSSION

Changes in the phytochemical composition of olives during ripening have been noted previously,^5^, ^22^ including the fall in phenolics seen here with ripening in both cultivars. The chlorophyll and Jaen index confirm that olives from the two cultivars were at equivalent stages of maturity at harvest. Hence, differences across growth locations and variety were not due to differences in stage of maturity. In our work the three sites of differing altitude were selected to ensure similar agricultural practices were used, and the three locations were in the same region of Italy; however, it is of course possible that small differences across the three sites in addition to altitude contributed to the differences noted. Nevertheless, phenolic content in ‘Nocellara messinese’ olives was consistently higher when grown in Mongrassano (highest altitude location) compared to the other two locations at each stage of development. This agreed with previous work showing a positive correlation between altitude of olive tree growth and phenolic content.^13^ Combining all the non‐volatile phytochemical and physiological characteristics gave the best discrimination amongst olive samples with almost complete discrimination within each maturity stage. A complete analysis of this type is labor intensive, and three characteristics: relative abundance of lipids, water, and phenolics, accounted for most of the discriminatory power. However, alone each characteristic could not discriminate growth location or cultivar.

Differences in total phenolics of oils has also been reported across cultivars^33^, ^34^ and growth location of the olives.^5^, ^35^ Here total phenolics were consistently higher in oil from olives grown at the highest location, Mongrassano, although differences were not always significant, while there was no clear difference relating to growth location in tocopherol content. This contrasts with previous work^36^ showing a negative correlation between altitude and tocopherols but no effect of altitude on total phenolics. Moreover, here acidity was significantly higher in oils from olives grown at Mirto Crosia (lowest altitude), contrasting with previous work^14^ finding no difference in oil acidity for the same cv. grown at different locations. However different cultivars were included in these studies.

As shown previously,^16^ combining oil non‐VOC characteristics can enable growth locations for the same cultivar to be discriminated accurately, and just the fatty acid profile can also be an excellent discriminator amongst varieties.^33^ The high abundance of oleic and linoleic acids in the oils found here agrees with previous studies.^1^, ^4^ Lignoceric and eicosenoic (gadoleic) acid have also been found at low abundance in other studies^5^ and varied with cultivar. Here, their relative abundance discriminated oils by stage of olive maturity, olive growth location and cultivar. Growth across a similar altitude range was previously shown to affect both drupe development and fatty acid content of oil, but found no difference in the percentage content of eicosenoic or lignoceric acids.^37^ In contrast palmitic acid and arachidic acid differed, which were less important discriminators in our study. Eicosenoic acid did vary, however, across oils from different cultivars^33^ but was also affected by crop year. Thus, the effect of growth altitude on oil profiles may also differ by both cultivar and season. Here olives from only one season were analyzed, and it will be important to repeat the study in subsequent years, ideally with differing climatic conditions to assess whether the discriminatory power of the relative abundance of eicosenoic acid is sufficiently robust for use as a marker of geographical origin.

Oil VOCs detected here belonged to all the major families generally comprising the aroma of olive oil,^38^ although we also detected terpenes and VOCs containing sulfur. As previously reported, VOCs putatively identified here as (E/Z)‐3‐hexenal (C22, C23), hexanal (C40) and (E/Z)‐2‐hexenal (C10, C11) were found in the VOC profiles and indeed here were amongst the most abundant VOCs across all samples. However, 3‐methylbutan‐1‐ol reported in most European olive oils^38^ was not detected here.

Previously a drop in relative abundance of *trans*‐2‐hexenal was found in oil aroma^15^ with olive ripeness. Here, it was not possible to fully differentiate between the two 2‐hexenal isomers. However, the relative abundance of the VOC putatively identified as (Z/E)‐2‐hexenal (C11, likely the E, or *trans* isomer since the RI is slightly higher), fell with increasing ripeness, although not significantly when averaged out across both cultivars and growth locations. In contrast, previously, an increase in (E)‐2‐hexenal with harvesting time was found for cv. Nocellara del Belice,^39^ a closely related cv. to Nocellara messinese, which was not seen here. This may be due to differences in maturity staging, season or cultivation. The relative abundance of the VOC putatively identified as hexan‐1‐ol (C3) in the oil VOC profile fell with increasing olive ripeness, in agreement with previous work.^15^, ^39^ This fall was ascribed to an increase in LOX pathway activity.^15^

The most discriminatory VOCs in stage 4 oil from our study were putatively identified as hexanal (C40), considered a potent odorant in olive oils,^40^ methyl acetate (C26), and 3‐hexen‐1‐ol (C20). In oil from stage 2 olives, VOCs putatively identified as 2‐methyl‐1‐propanol (C7), and ethyl acetate (C36) also contributed substantially to discrimination. Two of these are C6 VOCs, and when VOCs were sampled using solid‐phase microextraction (SPME), related C6 VOCs were identified as good discriminators for cultivars.^34^ Hexanal, ethyl acetate, and 3‐hexen‐1‐ol were also detected previously in one study,^34^ but not methyl acetate or 2‐methyl‐1‐propanol. In contrast, another study^41^ identified all five of these VOCs over 24 cultivars. However, they found different VOCs as being most dissimilar across the cultivars. Another two studies, of olive oils from Italy^39^, ^42^ also identified VOC markers useful for cv. discrimination including hexanal, amongst others. Differences may be due to the cultivars studied or the method of VOC analysis. Again, further analysis in different seasons will be necessary to verify the robustness of the VOC markers identified here.

Previously, significant differences in the profiles of oil VOCs derived from the LOX pathway were not found when the same olive cv. was grown in different locations.^6^ However other studies^42^ found clear discrimination of VOC profiles from the oil of several different cultivars by growth location. Here the whole VOC profile was able to discriminate both between cultivars grown at the same location and the same cultivar grown at different locations, although discrimination amongst growth locations was better when oil was derived from a single olive ripeness stage, and there were interactions amongst cultivar and location. This indicates that further work is needed to assess their value as markers.

Activation of LOX pathway enzymes is an important component of the development of oil aroma, leading to formation of C6 VOCs such as hexanal and 3‐hexen‐1‐ol identified here as key discriminators of both growth location and cultivar. Indeed, the C6 content of VOC profiles can be indicative of the LOX pathways that are active. For example, as previously noted^6^ and confirmed here, the cultivar Carolea oil VOC profile does not contain (E)‐3‐hexenyl acetate but it does contain (E)‐3‐hexen‐1‐ol (C20) indicating that the alcohol acetyl transferase (AAT) converting this alcohol to (E)‐3‐hexenyl acetate is not active.^38^

## CONCLUSIONS

Overall, we showed that a small number of both VOCs (hexanal, methyl acetate and 3‐hexen‐1‐ol), and non‐VOCs (eicosenoic acid and eignoceric acid) may have potential as markers for discriminating oils across stages of olive maturity, cultivar, and growth location when used in combination. This may be of use to the industry in verifying geographical origin of olives and oils, and for single variety oils, the cultivar used. However, different markers may be needed for differentiating amongst different cultivars. Moreover, when different olive growth stages are combined discrimination is more challenging. Importantly, the robustness and resilience across different VOC analysis platforms, seasons, growth locations, and olive maturity will need further validation as well as analyses of oils subjected to full‐scale industrial processing.

## Supporting information


**Table S1** Non‐VOC characteristics of (A) olives, (B) olive oils (C) PerMANOVA analysis of all factors
**Table S2,** VOCs detected across all samples
**Table S3,** Relative abundance of each VOC for (A) olives and (B) olive oils
**Table S4,** Non‐parametric tests and PerMANOVA to assess differences across cultivar, location and stage for each fatty acid.
**Fig. S1,** Locations and climatic information on olive trees;
**Fig. S2,** Linear discriminant plots from CAP based on non‐VOC characters in olives;
**Fig. S3,** Linear discriminant plots from CAP based on VOCs in olives;
**Fig. S4,** non‐VOC characters of oils;
**Fig. S5,** Heat maps of fatty acid composition of oils;
**Fig. S6,** Linear discriminant plots from CAP based on non‐VOC characters in olive oil;
**Fig. S7** Linear discriminant plots from CAP based on VOCs in olive oil.
**Fig. S8,** Random Forest analysis of VOCs from stage 2 olive oil.
