# Supplementary material for: Identifying volatile and non‐volatile organic compounds to discriminate cultivar, growth location, and stage of ripening in olive fruits and oils
Source: J Sci Food Agric. 2022 Feb 17;102(11):4500–13. doi: 10.1002/jsfa.11805 (PMC9541169; doi:10.1002/jsfa.11805)
Supplement: Supplementary file 1 — Table S1 Non‐VOC characteristics of (A) olives, (B) olive oils (C) PerMANOVA analysis of all factors Table S2, VOCs detected across all samples Table S3, Relative abundance of each VOC for (A) olives and (B) olive oils Table S4, Non‐parametric tests and PerMANOVA to assess differences across cultivar, location and stage for each fatty acid. Fig. S1, Locations and climatic information on olive trees; Fig. S2, Linear discriminant plots from CAP based on non‐VOC characters in olives; Fig. S3, Linear discriminant plots from CAP based on VOCs in olives; Fig. S4, non‐VOC characters of oils; Fig. S5, Heat maps of fatty acid composition of oils; Fig. S6, Linear discriminant plots from CAP based on non‐VOC characters in olive oil; Fig. S7 Linear discriminant plots from CAP based on VOCs in olive oil. Fig. S8, Random Forest analysis of VOCs from stage 2 olive oil. [file JSFA-102-4500-s001.docx]

**SUPPLEMENTARY TABLES AND FIGURES**

**Identifying Volatile and Non-Volatile Organic Compounds for Discriminating Cultivar, Growth Location and Stage of Ripening in Olive Fruits and Oils.**

Maria Greco^1,2#^, Natasha Spadafora^2,3#^, Martin Shine^2^, Ann Smith^2^, Antonella Muto^1^, Innocenzo Muzzalupo^4^, Adriana Chiappetta^1^, Leonardo Bruno^1^, Carsten Müller^2^, Hilary Rogers^2*^, Beatrice M. Bitonti^1^.

***corresponding author:** Email: rogershj@cf.ac.uk

Tel: +44(0)2920876352

Fax: +44(0)2920874305

| **Supplementary Table S1A – Olive non-VOC characteristics** | | | | | | | | | | |
| --- | --- | --- | --- | --- | --- | --- | --- | --- | --- | --- |
|  | **Jaen Index** | **Fat %** | **Dry Residue %** | **Water %** | **Chlorphyll a (mg/g)** | **Chlorophyll b (mg/g)** | **Sum of chlorophyll a and b (mg/g)** | | **Phenols (mg/g)** | **Carbohydrate %** |
|  | **A1** | **A2** | **A3** | **A4** | **A5** | **A6** | **A7** | | **A8** | **A9** |
| **CROS1R1** | 4.53 | 7.64 | 9.12 | 9.70 | 9.22 | 9.35 | 9.28 | | 8.83 | 8.67 |
| **CROS1R2** | 4.80 | 7.76 | 9.08 | 9.75 | 8.45 | 8.32 | 8.39 | | 8.44 | 9.08 |
| **CROS1R3** | 4.24 | 7.51 | 9.17 | 9.65 | 10.00 | 10.00 | 10.00 | | 9.18 | 8.15 |
| **CMoOS1R1** | 4.27 | 9.01 | 9.49 | 9.31 | 8.02 | 8.32 | 8.16 | | 9.67 | 9.21 |
| **CMoOS1R2** | 3.92 | 9.11 | 9.54 | 9.26 | 8.86 | 7.84 | 8.39 | | 10.00 | 9.47 |
| **CMoOS1R3** | 4.53 | 8.90 | 9.45 | 9.36 | 7.07 | 8.77 | 7.93 | | 9.34 | 8.72 |
| **CMiOS1R1** | 5.06 | 8.48 | 9.05 | 9.77 | 7.56 | 8.32 | 7.93 | | 8.00 | 8.20 |
| **CMiOS1R2** | 5.31 | 8.37 | 9.10 | 9.72 | 6.55 | 8.77 | 7.70 | | 8.38 | 7.90 |
| **CMiOS1R3** | 4.80 | 8.60 | 9.01 | 9.82 | 8.45 | 7.34 | 7.93 | | 7.59 | 8.72 |
| **NROS1R1** | 4.80 | 6.83 | 8.87 | 9.95 | 8.09 | 7.52 | 7.82 | | 7.03 | 8.58 |
| **NROS1R2** | 4.53 | 6.97 | 8.91 | 9.91 | 8.86 | 7.84 | 8.39 | | 7.45 | 8.90 |
| **NROS1R3** | 5.06 | 6.69 | 8.82 | 10.00 | 7.07 | 6.79 | 6.94 | | 6.51 | 8.38 |
| **NMoOS1R1** | 4.53 | 8.89 | 9.60 | 9.19 | 8.02 | 9.61 | 8.82 | | 9.63 | 9.21 |
| **NMoOS1R2** | 4.24 | 8.78 | 9.64 | 9.14 | 7.07 | 9.20 | 8.16 | | 9.36 | 9.47 |
| **NMoOS1R3** | 4.80 | 9.00 | 9.55 | 9.24 | 8.86 | 10.00 | 9.43 | | 9.90 | 8.72 |
| **NMiOS1R1** | 4.94 | 7.81 | 8.90 | 9.93 | 6.55 | 7.84 | 7.20 | | 6.16 | 7.80 |
| **NMiOS1R2** | 5.31 | 7.69 | 8.85 | 9.97 | 7.56 | 8.32 | 7.93 | | 5.55 | 8.25 |
| **NMiOS1R3** | 4.80 | 7.93 | 8.94 | 9.88 | 5.35 | 7.34 | 6.38 | | 6.65 | 7.38 |
| **CROS2R1** | 4.80 | 7.91 | 9.19 | 9.63 | 7.56 | 7.34 | 7.45 | | 7.49 | 7.90 |
| **CROS2R2** | 5.06 | 8.04 | 9.15 | 9.68 | 6.55 | 6.79 | 6.67 | | 7.93 | 8.44 |
| **CROS2R3** | 4.53 | 7.80 | 9.24 | 9.58 | 8.45 | 7.84 | 8.16 | | 7.06 | 7.21 |
| **CMoOS2R1** | 4.83 | 8.72 | 9.12 | 9.70 | 6.55 | 8.32 | 7.45 | | 9.18 | 9.38 |
| **CMoOS2R2** | 5.06 | 8.61 | 9.17 | 9.66 | 7.56 | 7.84 | 7.70 | | 9.55 | 9.92 |
| **CMoOS2R3** | 4.53 | 8.83 | 9.07 | 9.75 | 5.35 | 7.34 | 6.38 | | 8.75 | 9.17 |
| **CMiOS2R1** | 5.84 | 9.19 | 9.31 | 9.50 | 7.07 | 7.84 | 7.45 | | 7.31 | 8.76 |
| **CMiOS2R2** | 5.99 | 9.29 | 9.36 | 9.45 | 8.02 | 7.34 | 7.70 | | 7.72 | 9.21 |
| **CMiOS2R3** | 5.55 | 9.08 | 9.27 | 9.55 | 5.98 | 8.32 | 7.20 | | 6.80 | 8.46 |
| **NROS2R1** | 4.94 | 7.40 | 9.04 | 9.78 | 7.07 | 7.34 | 7.20 | | 6.32 | 8.20 |
| **NROS2R2** | 5.31 | 7.53 | 9.09 | 9.74 | 5.98 | 7.84 | 6.94 | | 6.81 | 7.92 |
| **NROS2R3** | 4.80 | 7.26 | 8.99 | 9.83 | 8.02 | 6.79 | 7.45 | | 5.86 | 8.69 |
| **NMoOS2R1** | 5.01 | 8.57 | 9.29 | 9.52 | 7.56 | 8.77 | 8.16 | | 9.24 | 8.76 |
| **NMoOS2R2** | 4.80 | 8.66 | 9.25 | 9.57 | 6.55 | 8.32 | 7.45 | | 8.86 | 9.23 |
| **NMoOS2R3** | 5.31 | 8.43 | 9.34 | 9.47 | 8.45 | 9.20 | 8.82 | | 9.64 | 8.51 |
| **NMiOS2R1** | 5.43 | 8.76 | 9.38 | 9.43 | 5.35 | 6.79 | 6.09 | | 3.83 | 8.85 |
| **NMiOS2R2** | 5.31 | 8.87 | 9.33 | 9.48 | 3.78 | 7.34 | 5.77 | | 4.27 | 9.42 |
| **NMiOS2R3** | 5.77 | 8.66 | 9.42 | 9.38 | 6.55 | 6.20 | 6.38 | | 3.06 | 8.85 |
| **Supplementary Table S1A –Olive non-VOC characteristics cont.d** | | | | | | | | | | |
|  |  |  |  |  |  |  |  | |  |  |
| **CROS4R1** | 7.16 | 9.17 | 9.96 | 8.76 | 5.35 | 5.55 | 5.44 | | 7.32 | 8.58 |
| **CROS4R2** | 7.34 | 9.06 | 10.00 | 8.71 | 3.78 | 4.80 | 4.30 | | 7.80 | 8.99 |
| **CROS4R3** | 6.98 | 9.27 | 9.92 | 8.81 | 6.55 | 6.20 | 6.38 | | 6.84 | 8.39 |
| **CMoOS4R1** | 9.41 | 9.91 | 9.95 | 8.77 | 4.63 | 8.32 | 6.67 | | 7.90 | 9.47 |
| **CMoOS4R2** | 9.61 | 9.81 | 10.00 | 8.71 | 3.78 | 7.84 | 6.09 | | 8.26 | 10.00 |
| **CMoOS4R3** | 9.34 | 10.00 | 9.91 | 8.82 | 5.35 | 8.77 | 7.20 | | 7.38 | 9.17 |
| **CMiOS4R1** | 9.71 | 9.40 | 9.31 | 9.50 | 3.78 | 4.80 | 4.30 | | 6.37 | 8.20 |
| **CMiOS4R2** | 9.87 | 9.50 | 9.36 | 9.45 | 2.67 | 3.92 | 3.33 | | 6.81 | 8.44 |
| **CMiOS4R3** | 10.00 | 9.30 | 9.27 | 9.55 | 4.63 | 5.55 | 5.09 | | 5.92 | 7.95 |
| **NROS4R1** | 7.16 | 8.90 | 9.66 | 9.12 | 3.78 | 3.92 | 3.85 | | 6.12 | 7.80 |
| **NROS4R2** | 7.34 | 8.79 | 9.61 | 9.17 | 2.67 | 2.77 | 2.72 | | 6.55 | 8.00 |
| **NROS4R3** | 6.98 | 9.01 | 9.70 | 9.07 | 4.63 | 4.80 | 4.71 | | 5.73 | 7.54 |
| **NMoOS4R1** | 8.09 | 9.14 | 9.40 | 9.41 | 4.63 | 7.84 | 6.38 | | 8.30 | 8.85 |
| **NMoOS4R2** | 8.01 | 9.24 | 9.35 | 9.46 | 3.78 | 7.34 | 5.77 | | 8.66 | 9.03 |
| **NMoOS4R3** | 8.32 | 9.03 | 9.44 | 9.36 | 5.35 | 8.32 | 6.94 | | 7.86 | 8.48 |
| **NMiOS4R1** | 7.97 | 8.89 | 9.46 | 9.34 | 4.63 | 4.80 | 4.71 | | 3.64 | 8.10 |
| **NMiOS4R2** | 7.84 | 8.78 | 9.41 | 9.39 | 3.78 | 3.92 | 3.85 | | 4.02 | 8.53 |
| **NMiOS4R3** | 8.16 | 9.00 | 9.51 | 9.29 | 5.35 | 5.55 | 5.44 | | 3.18 | 7.90 |
|  |  |  |  |  |  |  |  | |  |  |
|  |  |  |  |  |  |  |  | |  |  |
| **Key to sample names:** | |  |  |  |  |  |  | |  |  |
| **C** | Carolea | |  |  | **S1** | Stage 1 |  | |  |  |
| **N** | Nocellara | |  |  | **S2** | Stage 2 |  | |  |  |
| **Mi** | Mirto Crosia | |  |  | **S4** | Stage 4 |  | |  |  |
| **Mo** | Mongrassano | |  |  | **R1** | Replicate 1 | |  |  |  |
| **O** | Olive |  |  |  | **R2** | Replicate 2 |  | |  |  |
| **R** | Rende |  |  |  | **R3** | Replicate 3 |  | |  |  |
|  |  |  |  |  |  |  |  | |  |  |
| **Example:** |  |  |  |  |  |  |  | |  |  |
| **CMoOS1R1** | Carolea, Mongrassano, Olive, Stage 1, Replicate 1 | | | | | | | |  |  |

**Supplementary Table S1B – Oil non-VOC characteristics**

**Supplementary Table S1C**

**PerMANOVA analysis of significant differences for location, cultivar, maturity stage, and interactions between the factors across all samples.**

|  | **Location** | **Cultivar** | **Stage** | **Location *Cultivar** | **Location * Stage** | **Cultivar *Stage** | **Location * Cultivar* Stage** |
| --- | --- | --- | --- | --- | --- | --- | --- |
| **Olive characters** |  |  |  |  |  |  |  |
| R^2^ | 0.209 | 0.042 | 0.517 | 0.045 | 0.054 | 0.009 | 0.029 |
| P | 0.001 | 0.001 | 0.001 | 0.001 | 0.001 | ns | 0.015 |
| **Olive VOCs** |  |  |  |  |  |  |  |
| R^2^ | 0.066 | 0.024 | 0.327 | 0.063 | 0.086 | 0.021 | ns |
| P | 0.003 | 0.052 | 0.001 | ns | 0.011 | ns | ns |
| **Oil FAs** |  |  |  |  |  |  |  |
| R^2^ | 0.267 | 0.229 | 0.163 | 0.171 | 0.108 | 0.006 | 0.035 |
| P | 0.001 | 0.001 | 0.001 | 0.001 | 0.001 | 0.001 | 0.001 |
| **Oil characters** |  |  |  |  |  |  |  |
| R^2^ | 0.385 | 0.150 | 0.161 | 0.128 | 0.088 | 0.009 | 0.038 |
| P | 0.001 | 0.001 | 0.001 | 0.001 | 0.001 | 0.001 | 0.001 |
| **Oil VOCs** |  |  |  |  |  |  |  |
| R2 | 0.191 | 0.109 | 0.050 | 0.158 | 0.120 | 0.050 | ns |
| P | 0.001 | 0.001 | 0.001 | 0.001 | 0.001 | 0.001 | 0.001 |

*Includes all non-volatile characters.

**Supplementary Table S3A Square root of the relative abundance of each olive VOC to the grand total for each sample**

**Supplementary Table S3B Square root of the relative abundance of each olive oil VOC to the grand total for each sample**

**Supplementary Table S4A:** Non-parametric tests to assess differences across cultivar, location and stage for each fatty acid.

**Supplementary Table S4B PerMANOVA analysis of significant differences in the fatty acid composition across oil samples**

| **Fatty acid** | **Cultivar (C)** | **R2** | **Location (L)** | **R2** | **Stage (S)** | **R2** | **interactions amongst factors** | **R2** |
| --- | --- | --- | --- | --- | --- | --- | --- | --- |
| C 14:0 (Myristic acid) % | ns | n/a | 0.006 | 0.158 | 0.05 | 0.068 | CV/S +L/S | 0.09, 0.22 |
| C16:0 (Palmitic acid) % | 0.001 | 0.29 | 0.001 | 0.229 | ns | n/a | ns | n/a |
| C16:1 sum (Palmitoleic acid) % | 0.001 | 0.29 | 0.003 | 0.228 | 0.043 | 0.056 | ns | n/a |
| C17:0 (Margaric acid) % | 0.001 | 0.29 | 0.001 | 0.228 | 0.044 | 0.056 | ns | n/a |
| C17:1 (Margaroleic acid) % | 0.001 | 0.29 | 0.002 | 0.228 | ns | n/a | ns | n/a |
| C18:0 (Stearic acid) % | 0.001 | 0.29 | 0.001 | 0.228 | ns | n/a | ns | n/a |
| C18:1 (Oleic acid) % | 0.001 | 0.29 | 0.001 | 0.228 | 0.006 | 0.056 | ns | n/a |
| C18:2 (Linoleic acid) % | 0.001 | 0.29 | 0.001 | 0.228 | 0.046 | 0.056 | ns | n/a |
| C20:0 (Arachidic acid) % | 0.001 | 0.29 | 0.001 | 0.228 | 0.039 | 0.056 | ns | n/a |
| C18:3 (Linolenic acid) % | 0.001 | 0.29 | 0.001 | 0.228 | 0.043 | 0.056 | ns | n/a |
| C20:1 (Eicosenoic acid) % | 0.001 | 0.29 | 0.002 | 0.228 | 0.04 | 0.056 | ns | n/a |
| C22:0 (Behenic acid) % | 0.001 | 0.29 | 0.001 | 0.228 | ns | n/a | ns | n/a |
| C24:0 (Lignoceric acid) <0.2 % | 0.001 | 0.29 | 0.004 | 0.228 | ns | n/a | ns | n/a |

**A**

**Mongrassano**

**540 m ASL**

**Rende**

**225 m ASL**

**Mirto Crosia 8m ASL**

100 km

**B**


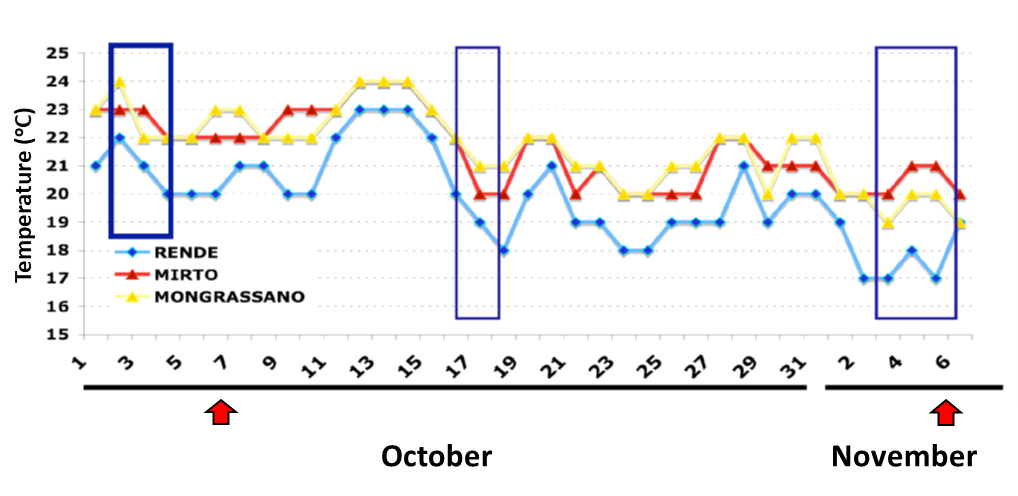


**Supplementary Figure S1 Locations and climatic information on olive trees A.** Map showing the three olive tree growth sites all in the Calabria region of Italy, and the altitude (as metres above sea level, ASL); **B** Temperature over a 37 day period starting 1 week before the first sampling time (red arrow) at each of the three locations. Second red arrow shows last sampling date.


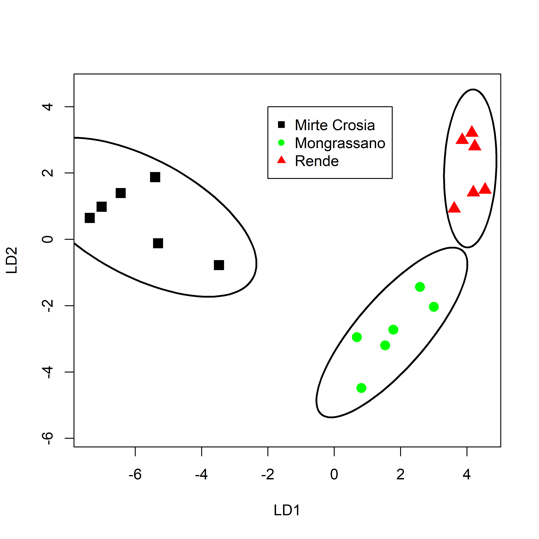

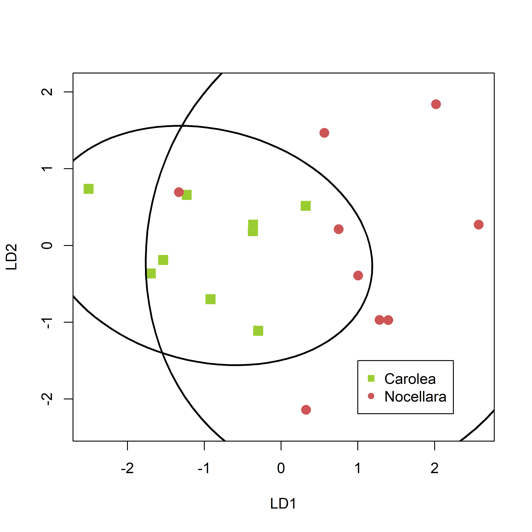


Olive stage 1


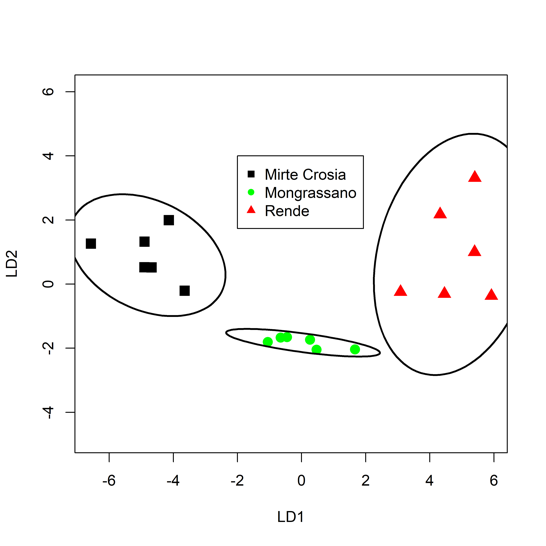

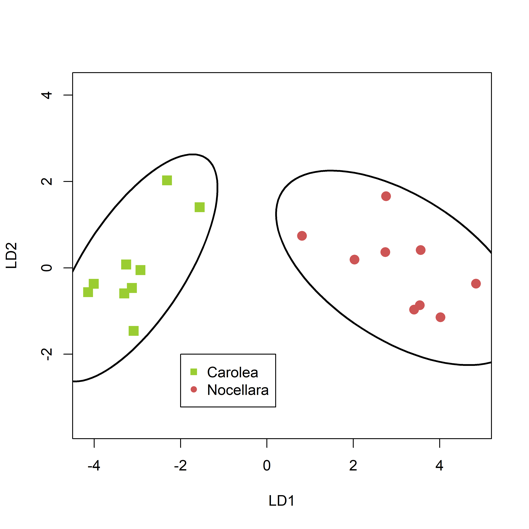

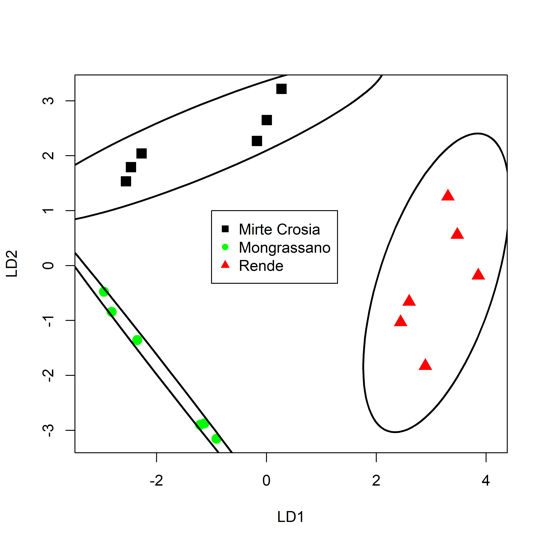

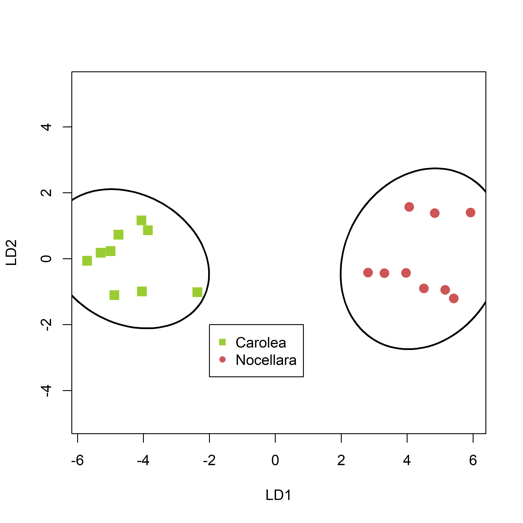


Olive stage 2

Olive stage 4

A

B

‘Carolea’

‘Nocellara messinese’


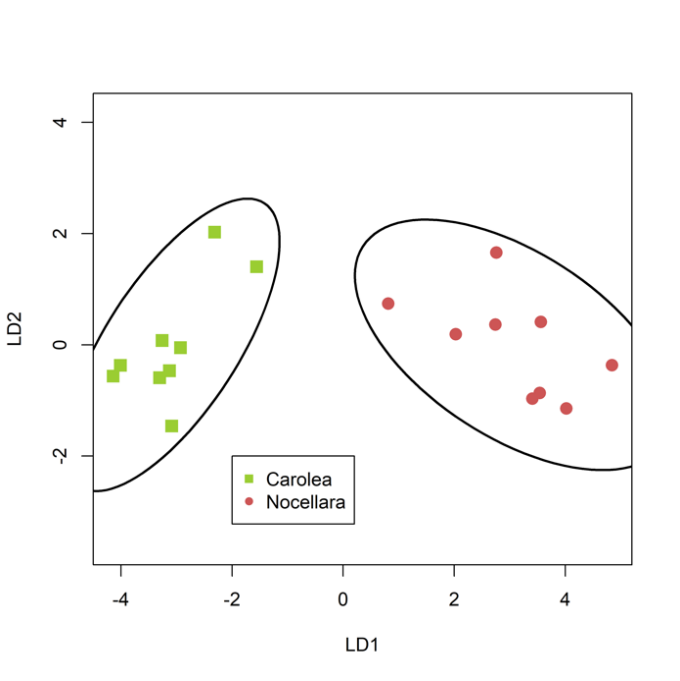


‘Carolea’

‘Nocellara messinese’


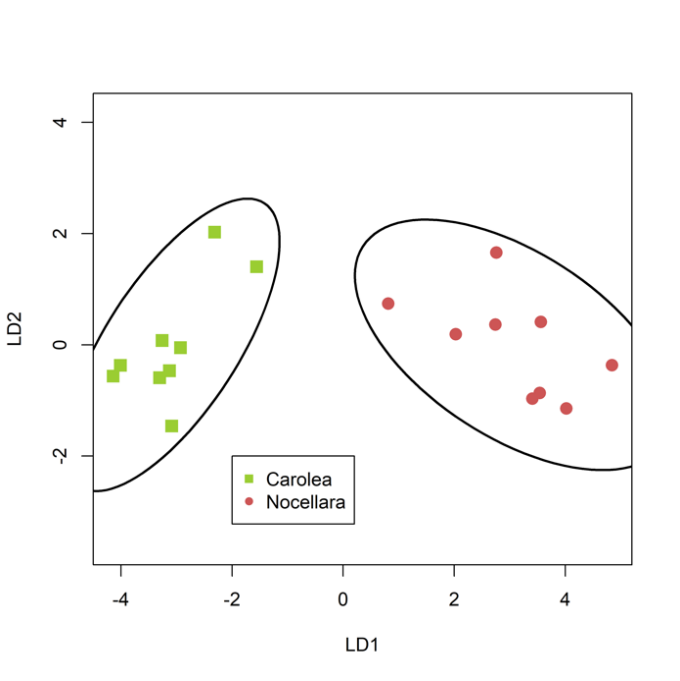


‘Carolea’

‘Nocellara messinese’


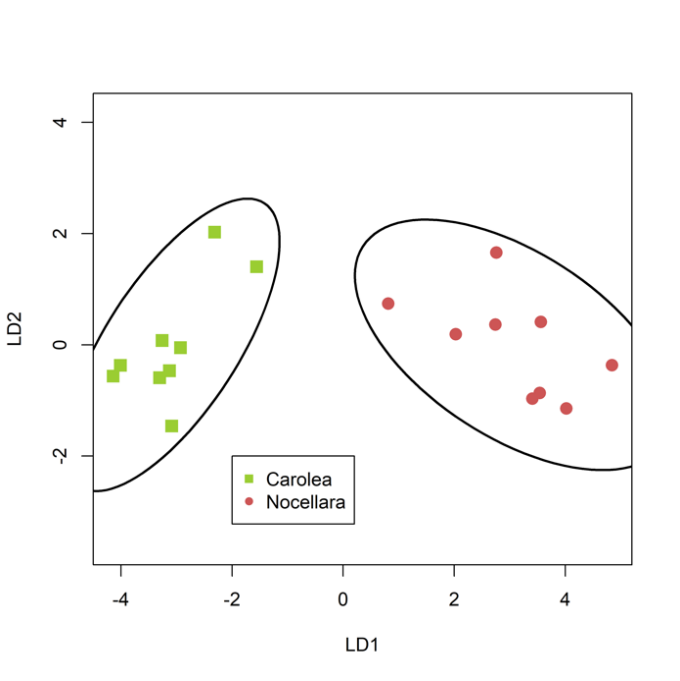


Mirto Crosia

Mongrassano

Rende


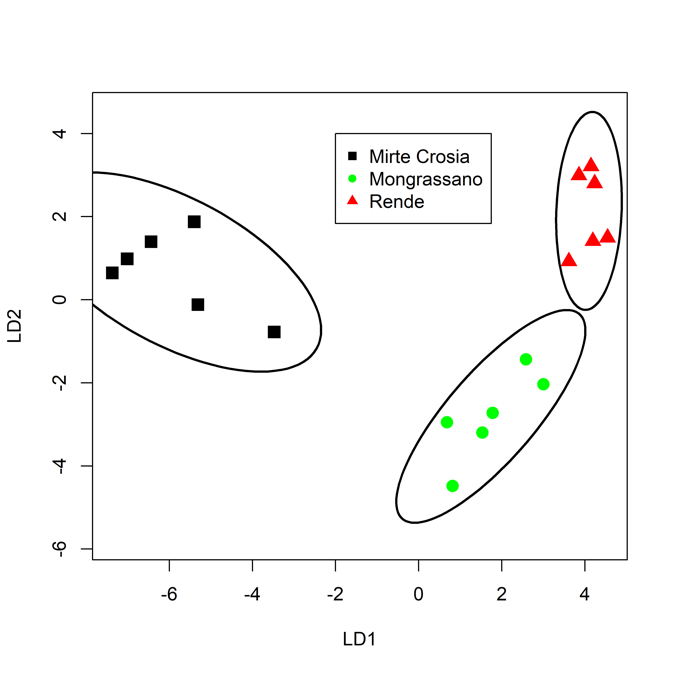


Mirto Crosia

Mongrassano

Rende


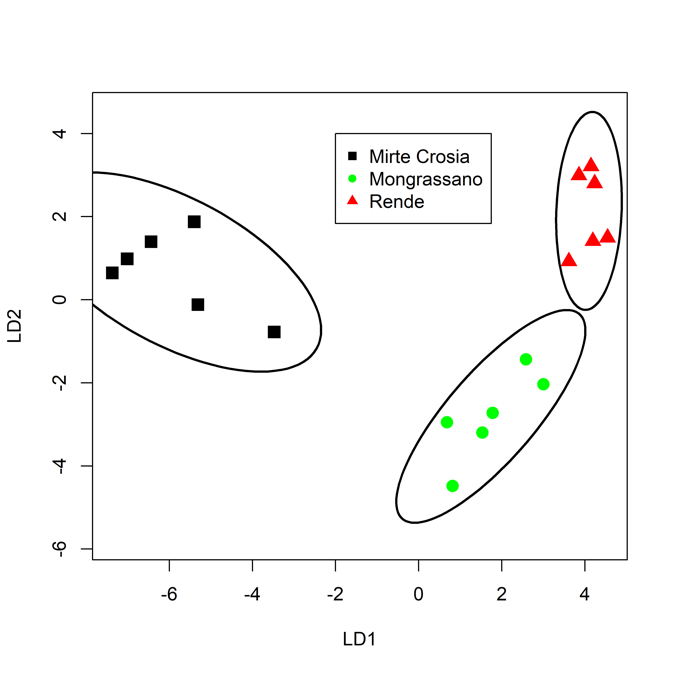


Mirto Crosia

Mongrassano

Rende


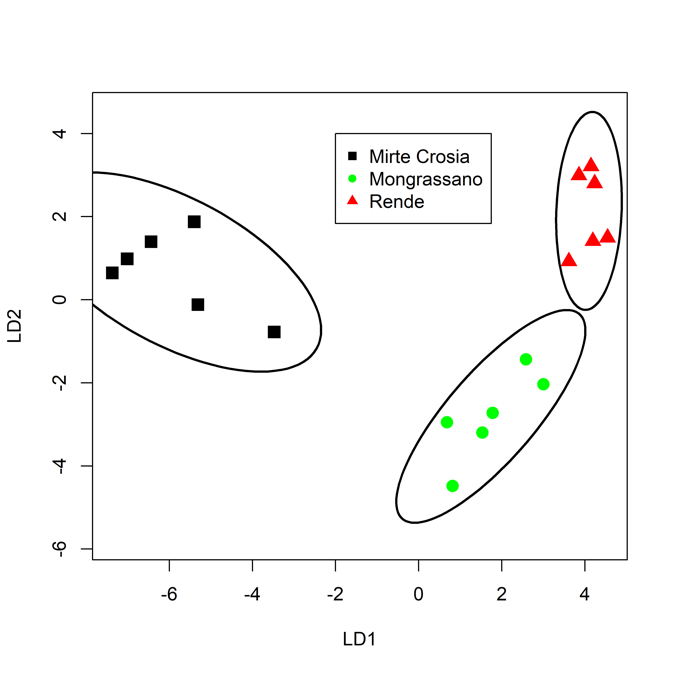


**Supplementary Figure S2. Linear discriminant plots from canonical analysis of principal coordinates (CAP) based on non-VOC characters in olives.** CAP models were produced for samples from **(A)** different locations (Mirto Crosia, Mongrassano, or Rende), **(B)** cultivar (‘Nocellara messinese’, or ‘Carolea’) for olives of three developmental stages (1, 2 and 4). Ellipses represent the 95% confidence interval (S.D.). Percentage of correct classifications: Stage 1 (A) 100% (*P* < 0.001), (B) 78% (*P* < 0.09); Stage 2 (A) 100% (*P* < 0.001), (B) 94% (*P* < 0.01); Stage 4 (A) 100 % (*P* < 0.001), (B) 100%. % (*P* < 0.001).

Olive: both cultivars and all locations

**Supplementary Figure S3. Linear discriminant plots from canonical analysis of principal coordinates (CAP) based on VOCs in olives.** CAP models were produced for samples from the three olive stages **(A)**, and different locations (Mirto Crosia, Mongrassano, or Rende) (**B, D, F**), and cultivar (‘Nocellara messinese’ or ‘Carolea’) (**C, E, G**) for olives of three developmental stages (1, 2 and 4). Ellipses represent the 95% confidence interval (S.D.). Percentage of correct classifications: (A) 77% (*P* = 0.09), (B) 83% (*P* = 0.82), (C) 72% (*P* = 0.31); (D) 39% (*P* = 0.98), (E) 72% (*P* = 0.03); (F) 56 % (*P* = 0.74), (G) 44% (*P* = 0.53).


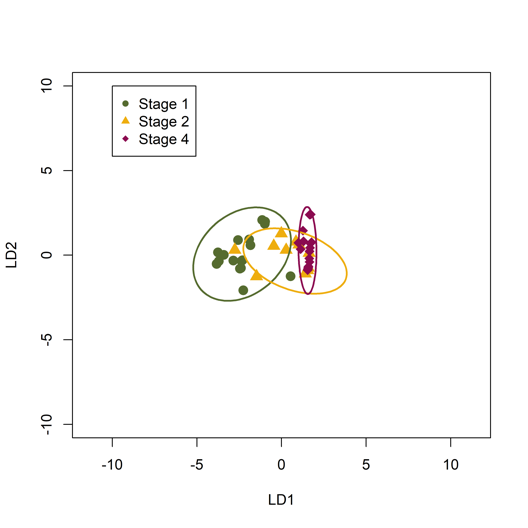

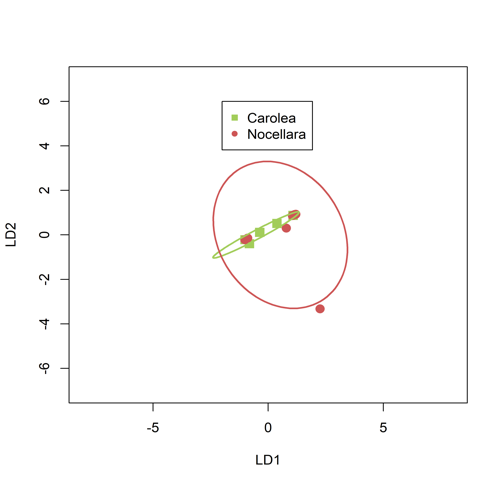


Olive stage 2

Olive stage 4

D

E

F

G

Olive stage 1

B

C

A


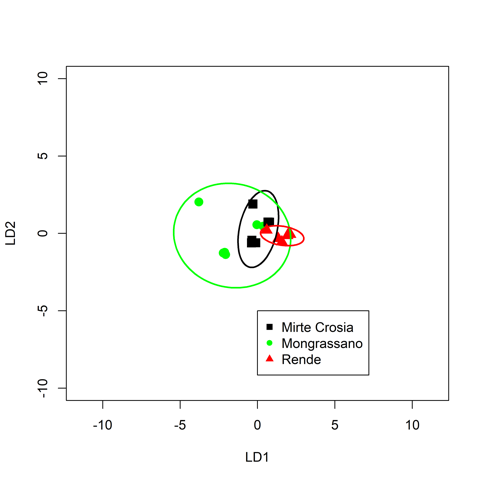


**Mirto Crosia**

**Mongrassano**

**Rende**


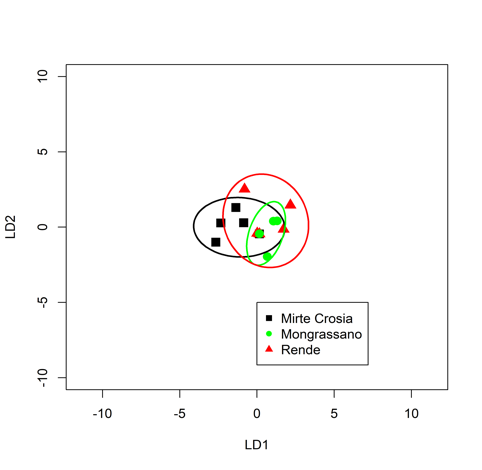


**Mirto Crosia**

**Mongrassano**

**Rende**


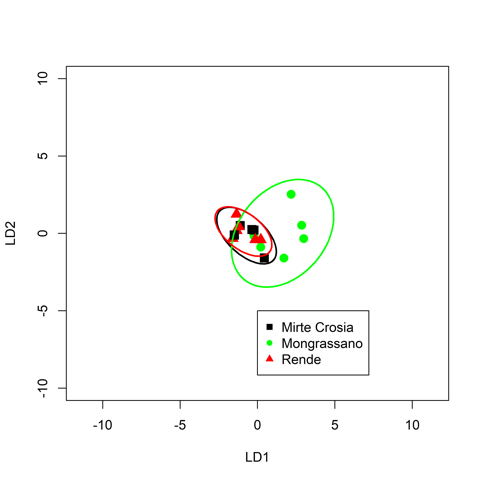


**Mirto Crosia**

**Mongrassano**

**Rende**


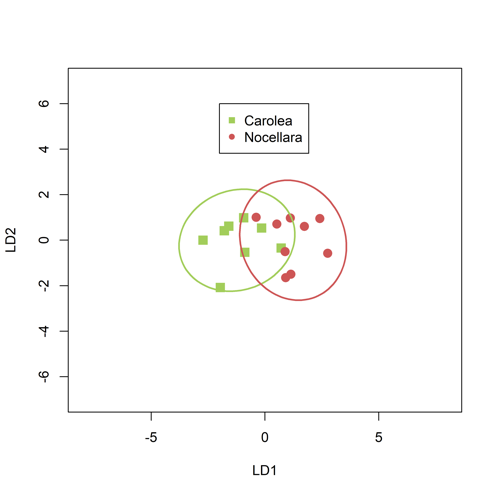

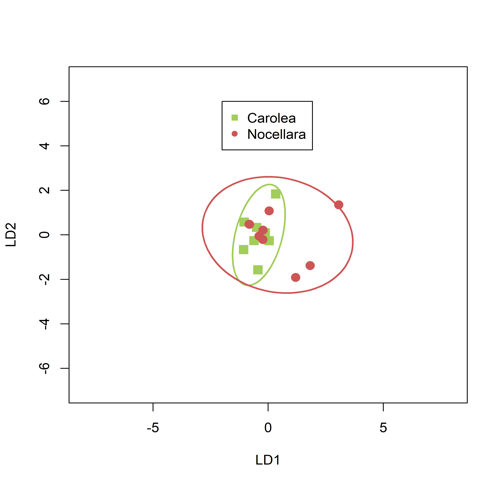


‘Carolea’

‘Nocellara messinese’


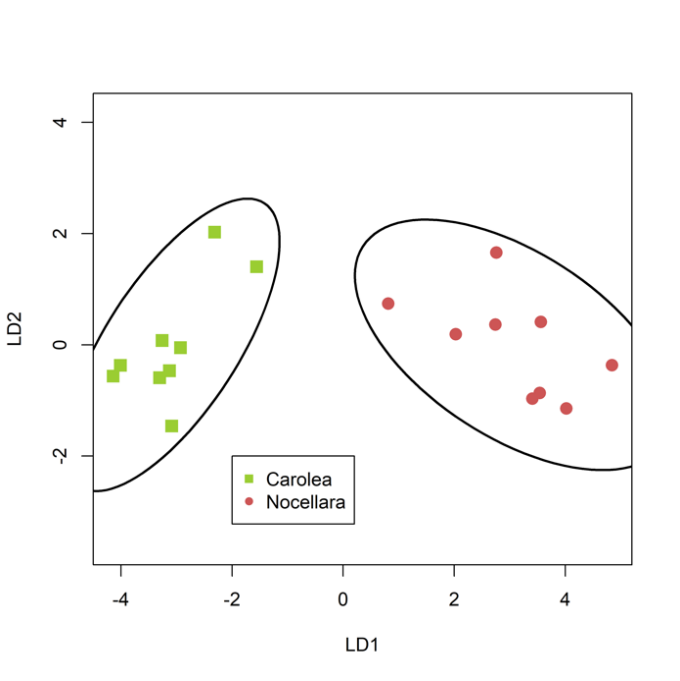


‘Carolea’

‘Nocellara messinese’


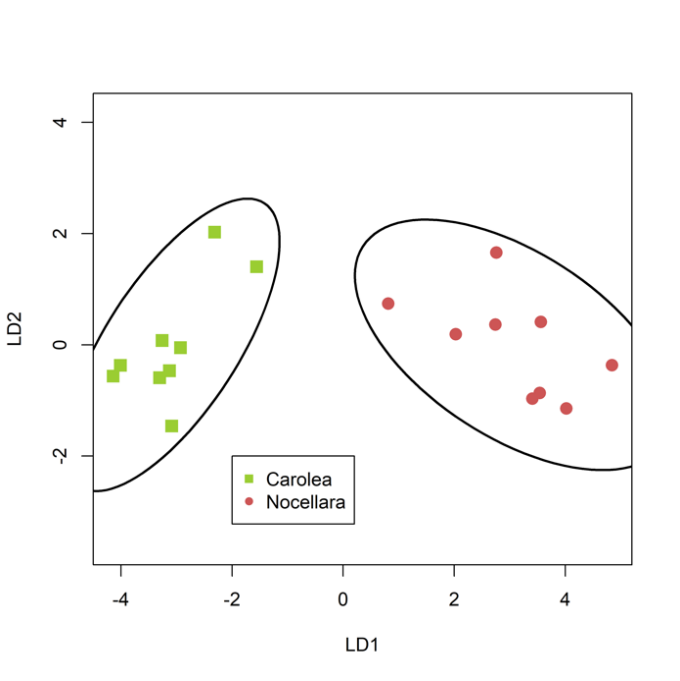


‘Carolea’

‘Nocellara messinese’


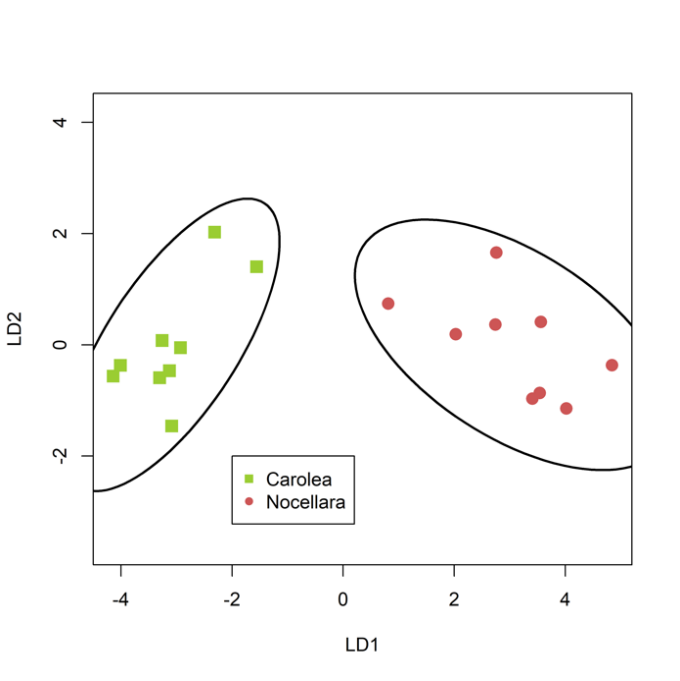

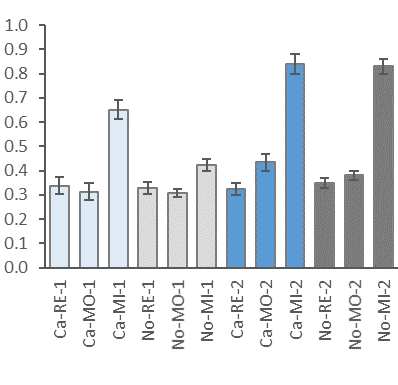

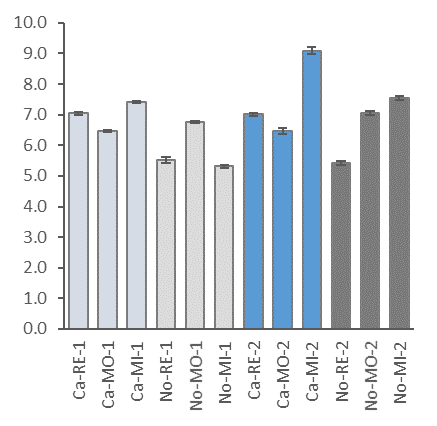


**A**

**B**

**C**


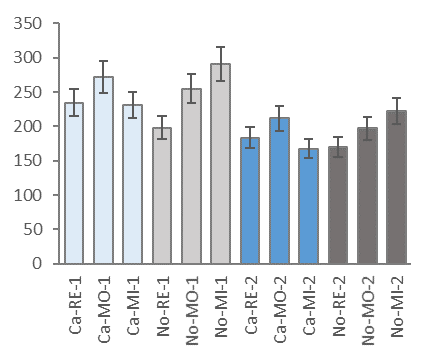

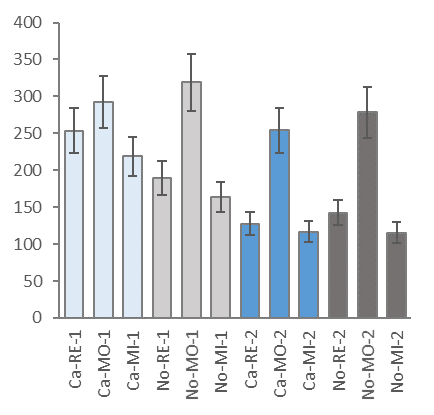


Acidity (% free oleic acid)

Tocopherols (ppm)

Peroxides (mEqO_2_/Kg)

Total phenolics asmg/g (caffeic acid)

bc

ab

cd

de

a

def

f

bc

f

ef

abc

f

bcd

ab

bcd

defg

abc

a

efg

def

g

fg

defg

cde

abcd

abce

acd

be

abcde

e

abcd

abe

d

be

abcd

cd

Ca-RE-2

Ca-MO-2

Ca-MI-2

No-RE-2

No-MO-2

No-MI-2

Ca-RE-4

Ca-MO-4

Ca-MI-4

No-RE-4

No-MO-4

No-MI-4

Ca-RE-2

Ca-MO-2

Ca-MI-2

No-RE-2

No-MO-2

No-MI-2

Ca-RE-4

Ca-MO-4

Ca-MI-4

No-RE-4

No-MO-4

No-MI-4

Ca-RE-2

Ca-MO-2

Ca-MI-2

No-RE-2

No-MO-2

No-MI-2

Ca-RE-4

Ca-MO-4

Ca-MI-4

No-RE-4

No-MO-4

No-MI-4

Ca-RE-2

Ca-MO-2

Ca-MI-2

No-RE-2

No-MO-2

No-MI-2

Ca-RE-4

Ca-MO-4

Ca-MI-4

No-RE-4

No-MO-4

No-MI-4

**D**

abc

abc

abc

c

c

bc

abc

ab

a

ab

a

ab

**Supplementary Figure S4. Non-VOC characters of oils** Acidity (A), peroxides (B) total tocopherols (C) and total phenolics (D) of oil from olives at two developmental stages (1 and 2) from two cultivars (Ca, ‘Carolea’, No, ‘Nocellara messinese’) grown in three different locations; (RE, Rende, MO, Mongrassano, MI, Mirto); n=3; +S.D. Letters indicate significant differences amongst growth locations (*P* < 0.05) based on one way ANOVA or Kruskall Wallis test followed by an LSD or Dunn’s test with Benjamini-Hochberg correction.


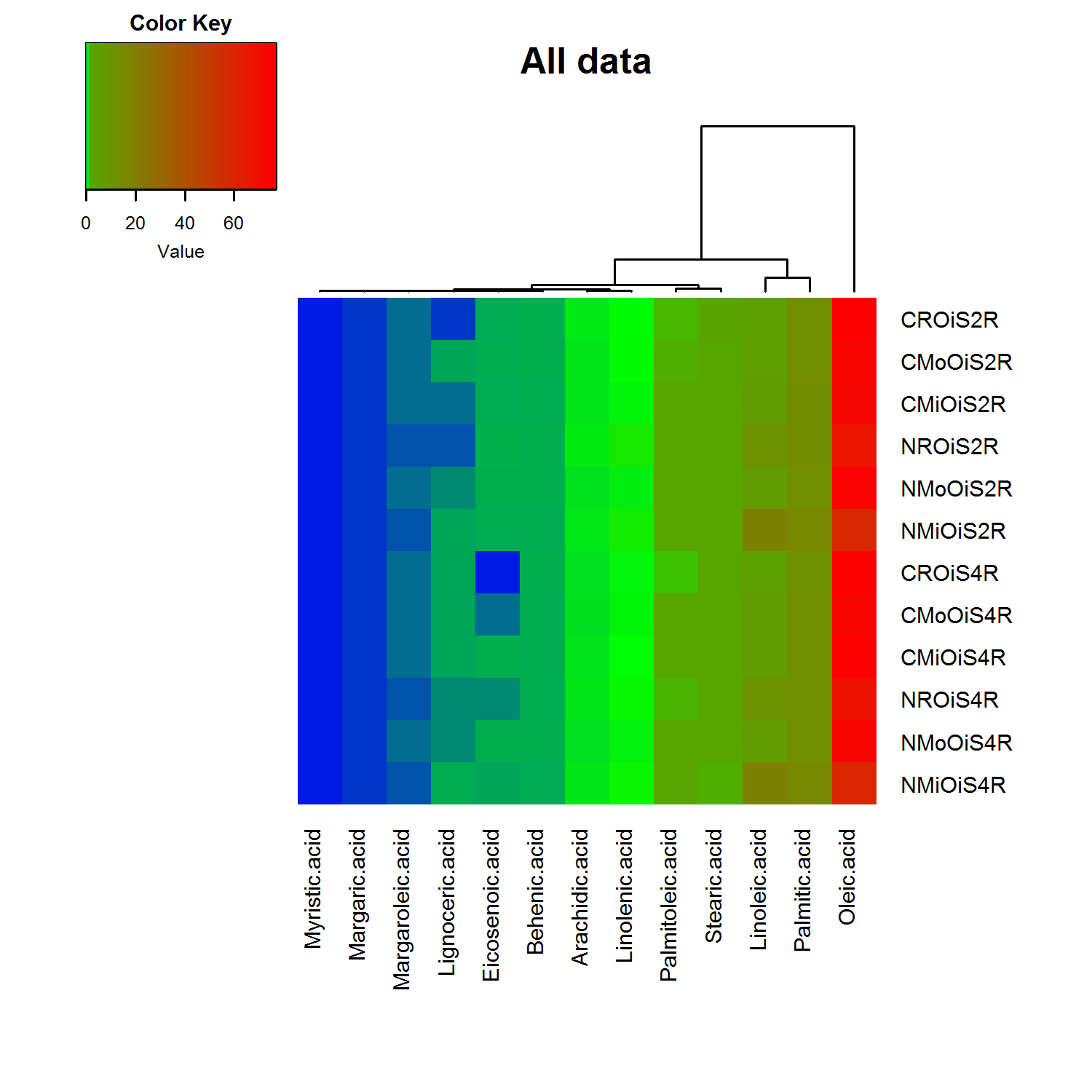

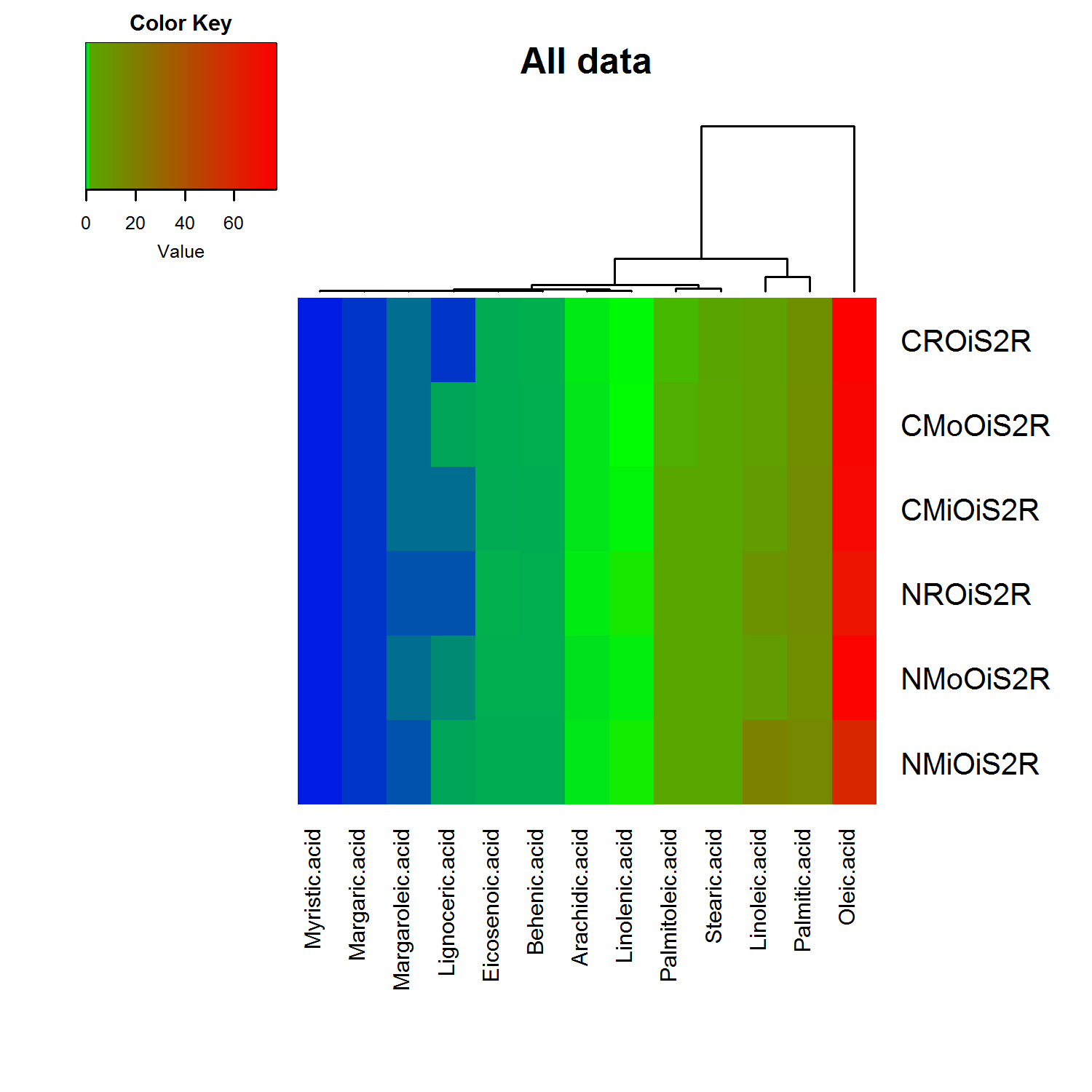

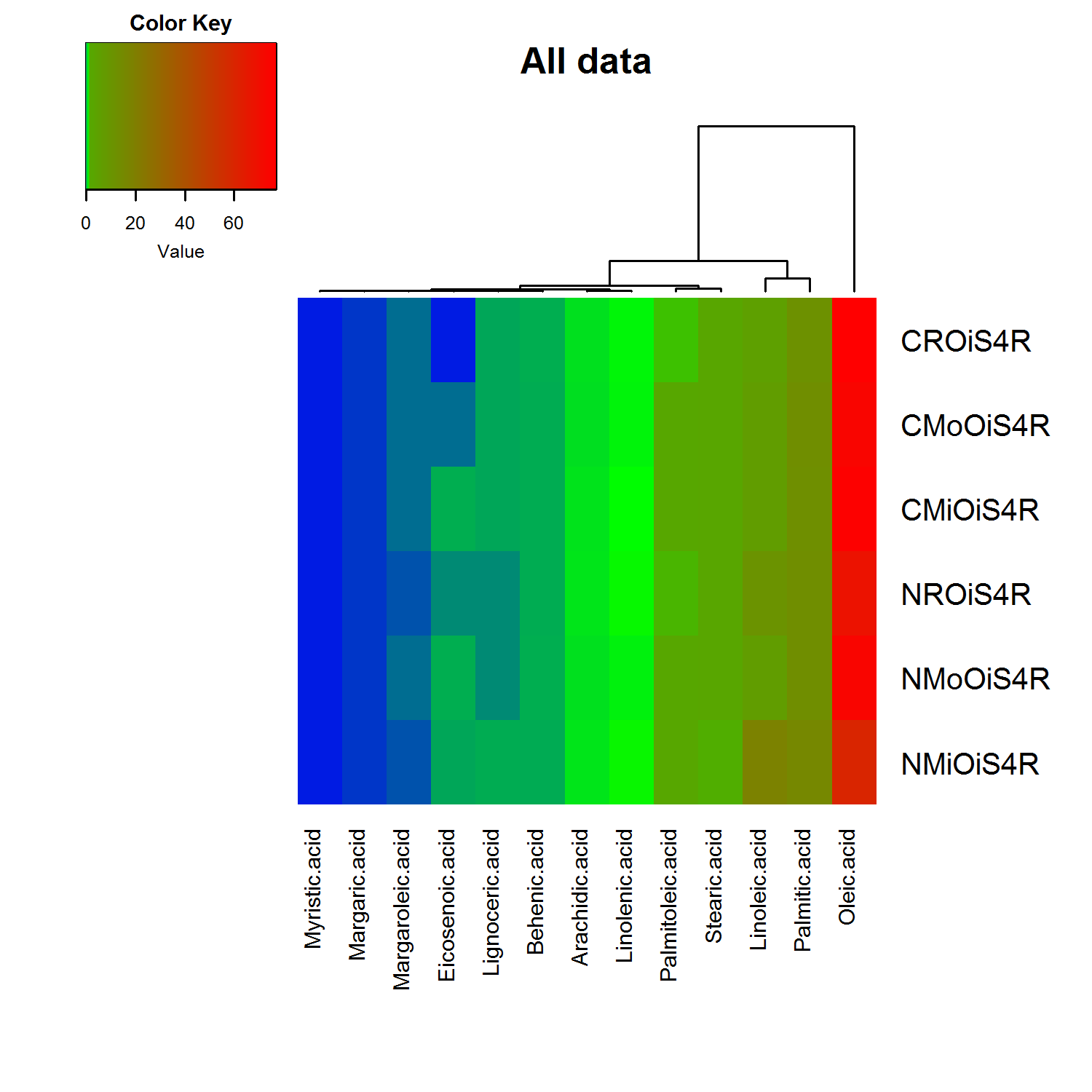


**(A) Stage 2 and 4**

**(B) Stage 2**

**(C) Stage 4**

**Supplementary Figure S5. Heat maps of fatty acid composition of oils** from two olive cultivars (‘Nocellara messinese’ (N) ) or ‘Carolea’ (C) ) grown at different locations (Mirto Crosia (Mi) , Mongrassano (Mo), or Rende (R) ), and of **(A)** two developmental stages (2 and 4) **(B)** only stage 2, **(C)** only stage 4. Means (n=3)


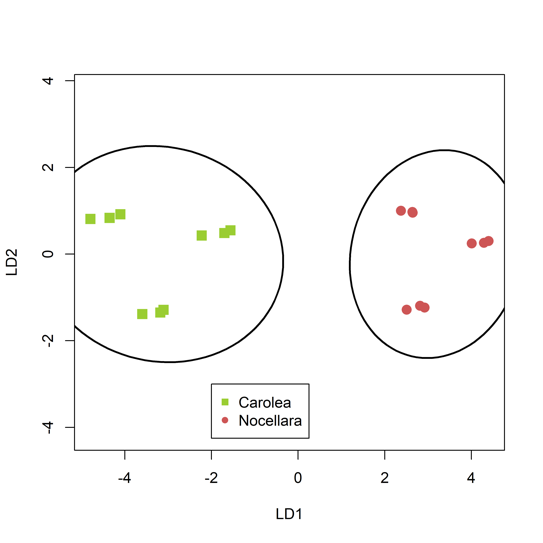

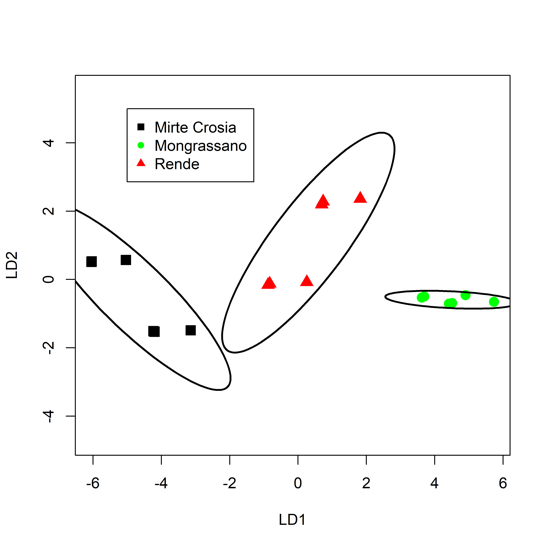

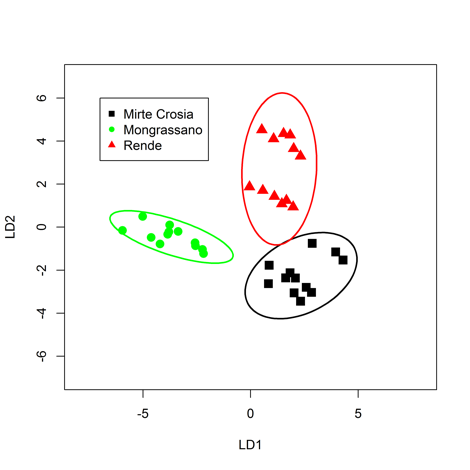

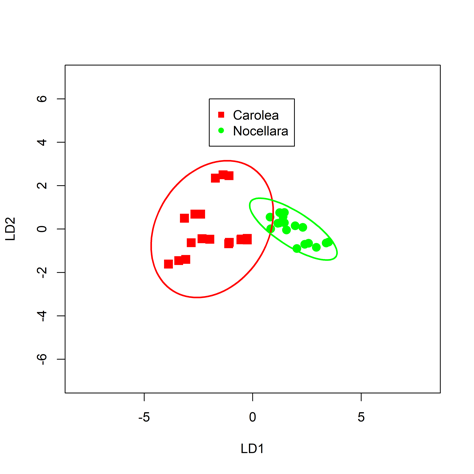

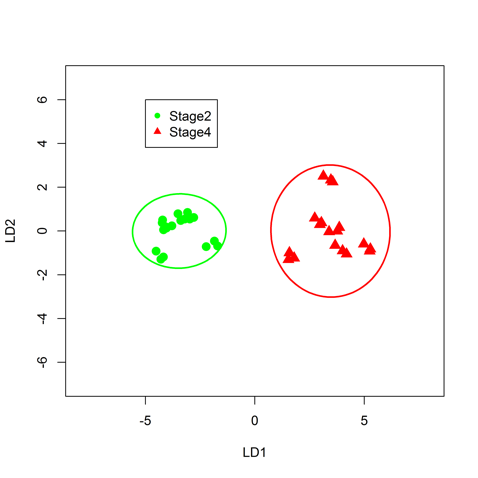


Oil: both cultivars and all locations

A

B

Oil stage 2

C

D

E

Oil stage 2 and 4

Oil stage 2 and 4

**Supplementary Figure S6. Linear discriminant plots from** **Canonical analysis of principal coordinates (CAP) based on non-VOC characters in olive oil.** CAP models were produced for oil derived from olives grown at (A,D) different locations (Mirto Crosia , Mongrassano, or Rende), (B,E) of different cultivars (Nocellara) or Carolea) (C) of two developmental stages (2 and 4) from all locations and both cultivars. Ellipses represent the 95% confidence interval (SD). Percentage of correct classifications: (A,B and C) 100% (*P* < 0.001), Stage 2 (D) 100% (*P* < 0.001), (E) 100% (*P* < 0.001), ; Stage 4 (D) 100% (*P* < 0.001), (E) 100% (*P* < 0.001).

Oil stage 2


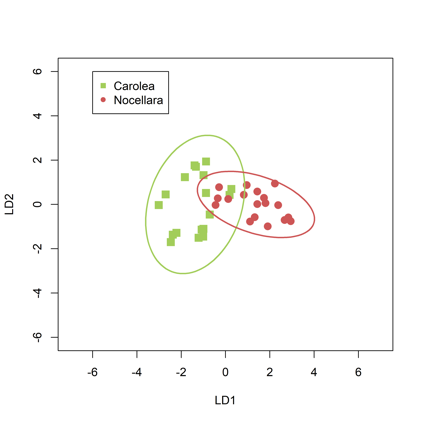

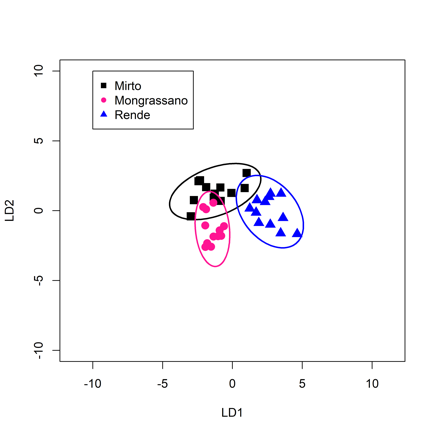

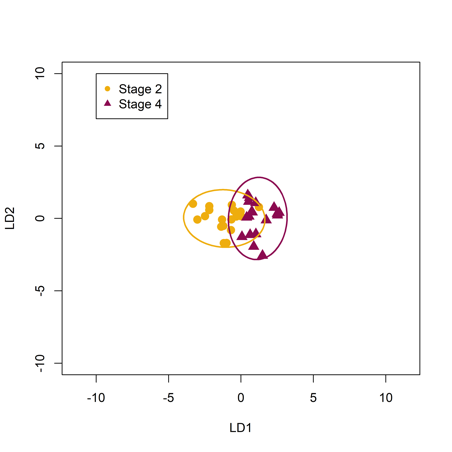


Oil stage 2 and 4

Oil stage 2 and 4

A

B

C


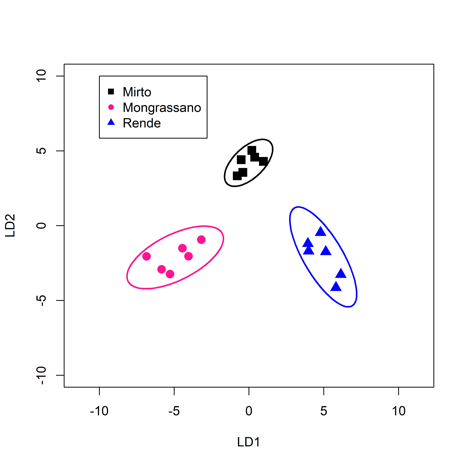


D


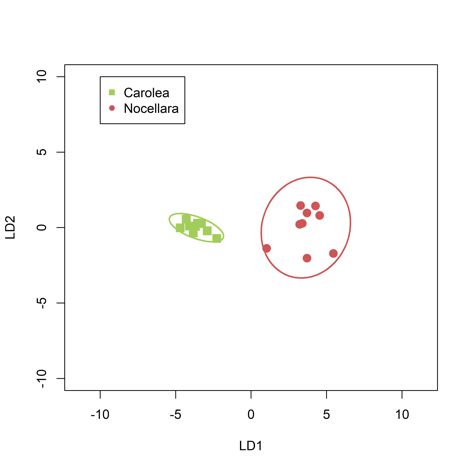


E


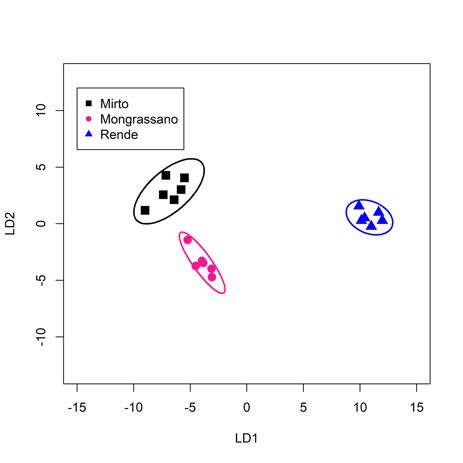

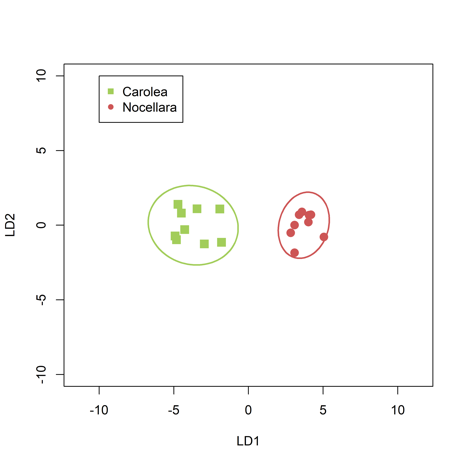


F

G

‘Carolea’

‘Nocellara messinese’


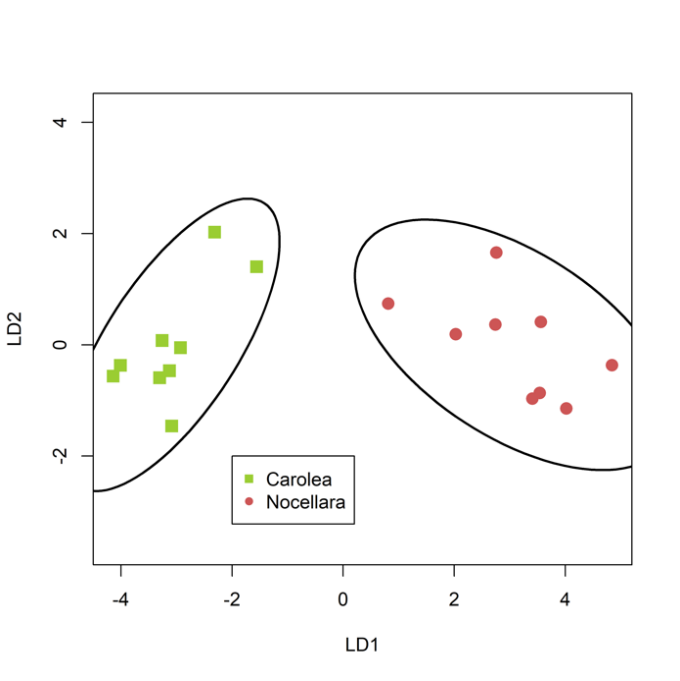


‘Carolea’

‘Nocellara messinese’


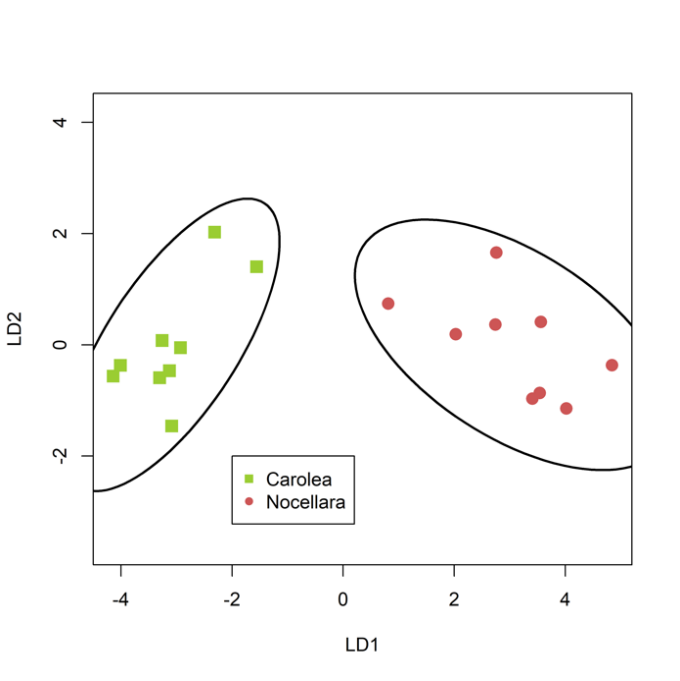


‘Carolea’

‘Nocellara messinese’


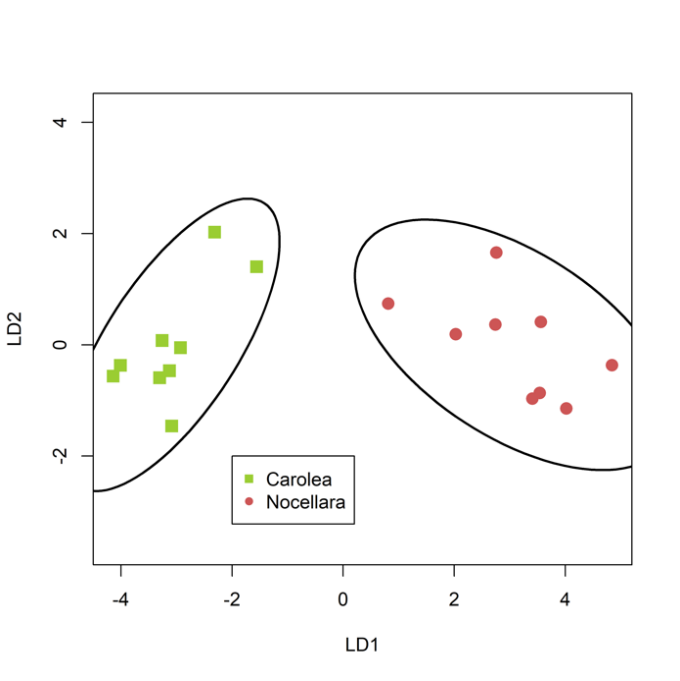


Mirto Crosia

Mongrassano

Rende


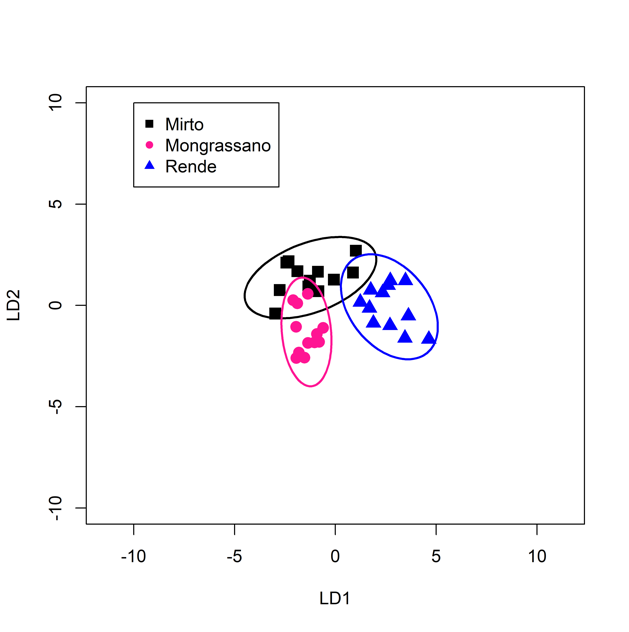


Mirto Crosia

Mongrassano

Rende


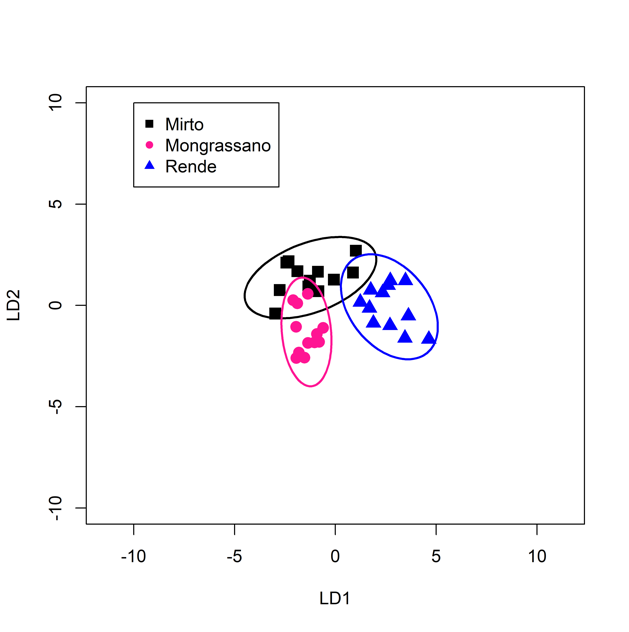


Mirto Crosia

Mongrassano

Rende


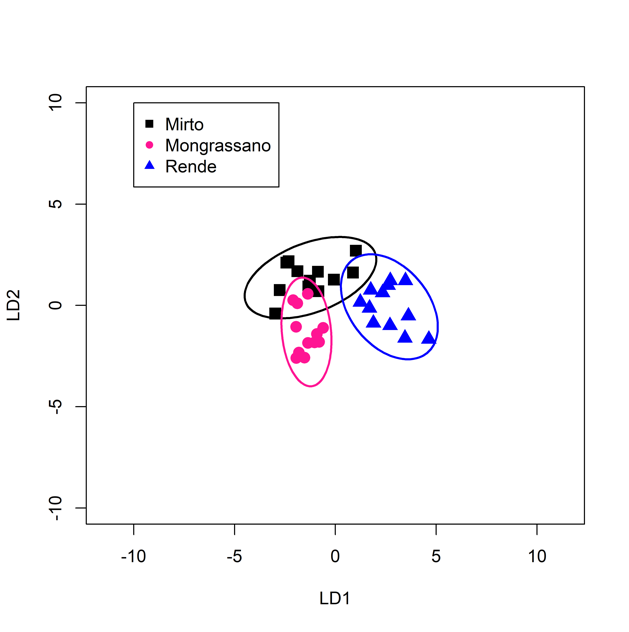


Oil: both cultivars and all locations

Oil stage 4

**Supplementary Figure S7. Linear discriminant plots from canonical analysis of principal coordinates (CAP) based on VOCs in olive oil.** CAP models were produced for oil derived from olives grown at **(A,D)** different locations (Mirto Crosia, Mongrassano, or Rende), **(B,E)** of different cultivars (‘Nocellara messinese’ or ‘Carolea’) **(C)** of two developmental stages (2 and 4) from all locations and both cultivars. Ellipses represent the 95% confidence interval (S.D.). Percentage of correct classifications: (A) 81% (*P* = 0.05); (B) 83% (*P* = 0.07); (C) 89% (*P* = 0.01); (D) 94% (*P* = 0.67); (E) 100% (*P* < 0.01); (F) 83% (*P* = 0.69); (G) 94% (*P* = 0.03).


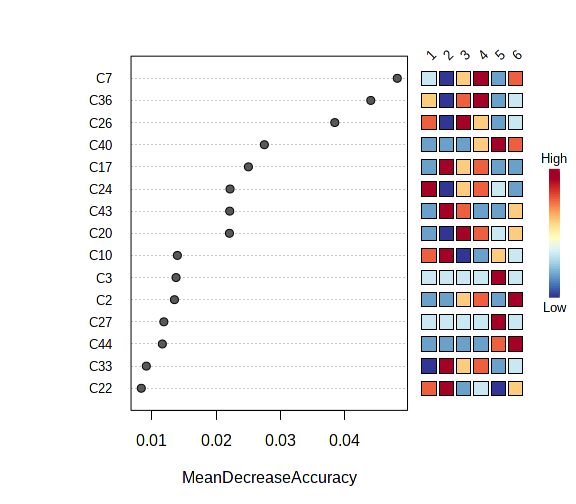


**A**

CMiOiS4R1

CMoOiS4R1

CROiS4R1

NMiOiS4R1

NMoOiS4R1

NROiS4R1

2-methyl-1-propanol

ethyl acetate

methyl acetate

hexanal

(5e)-3-ethyl-1,5-octadiene (e/z)

3-pentanone

Octane

3-hexen-1-ol

2-hexenal (e/z)

1-Hexanol

3-methyl-1-butanol

1,3-dimethylbenzene

Cyclobutyl hexyl oxalate

Ethylidenecyclopropane

3-Hexenal A


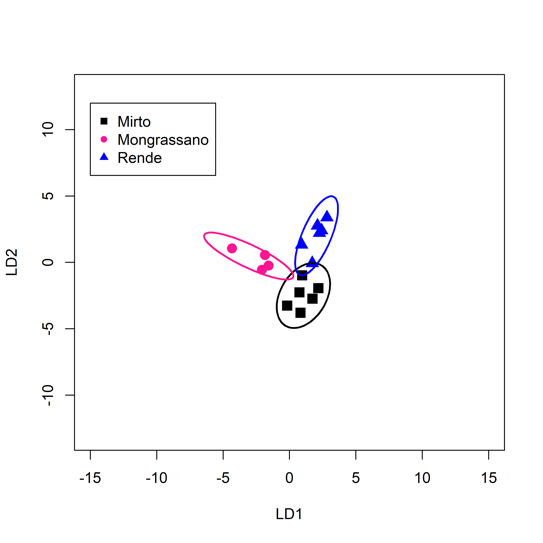

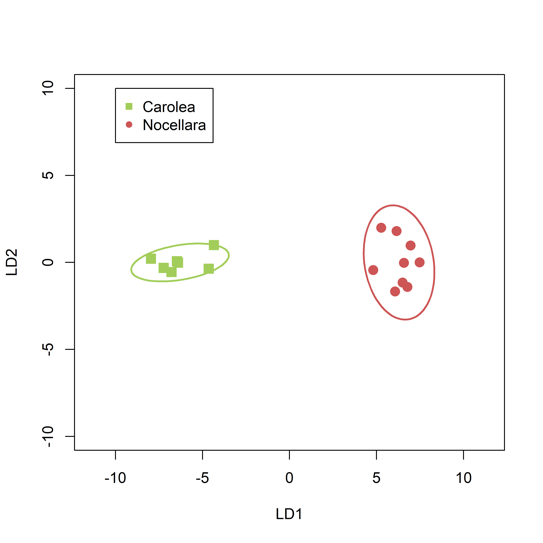


**B**

**C**


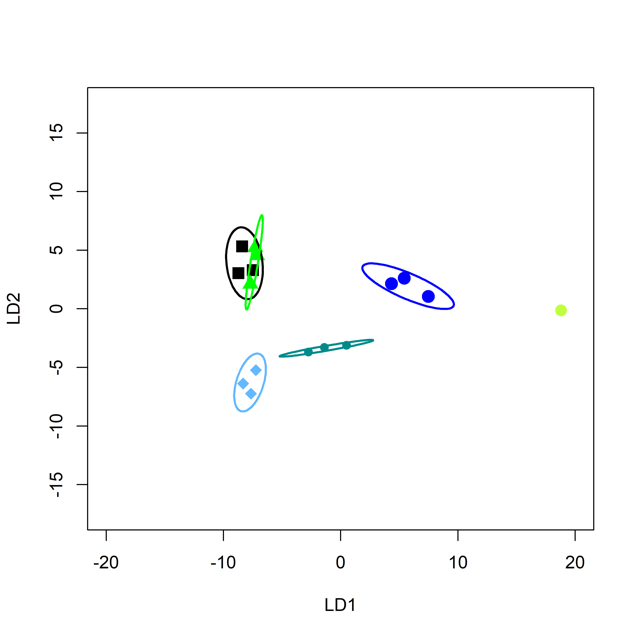


**D**

C, Mo

C, Re

N, Mi

N, Re

N, Mo

C, Mi

‘Carolea’

‘Nocellara messinese’


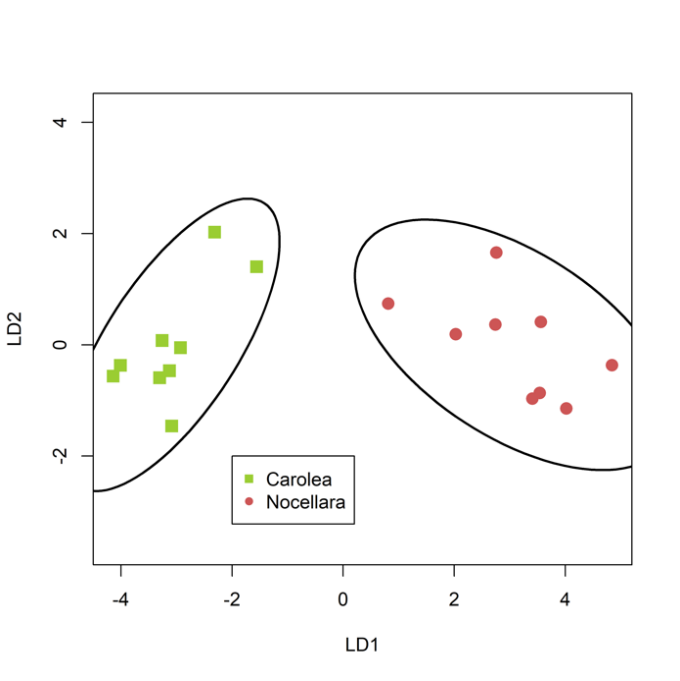


Mirto Crosia

Mongrassano

Rende


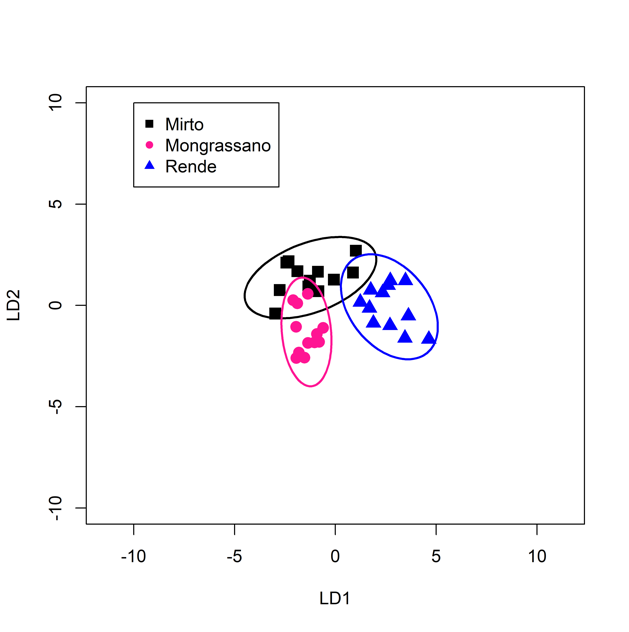

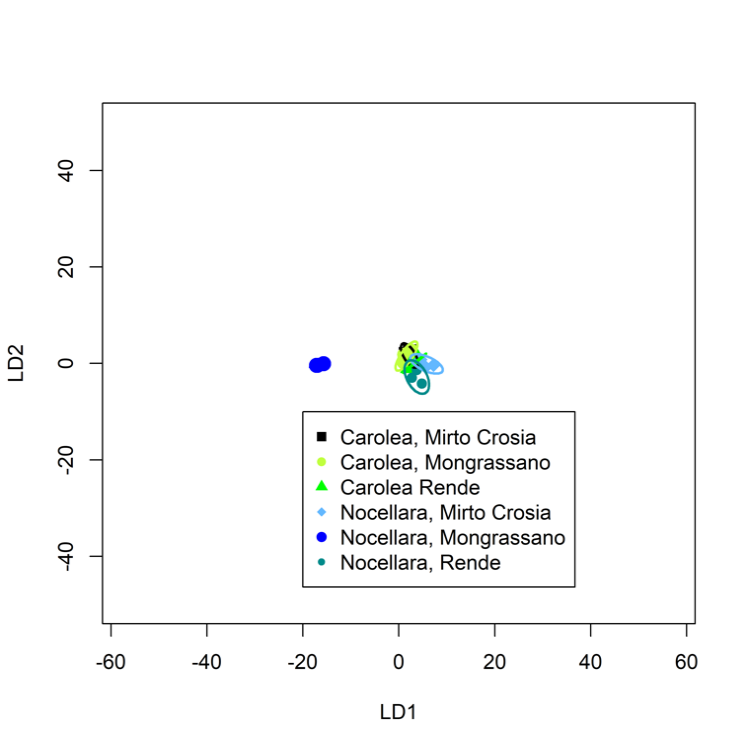


C,Mi

C, Mo

C, Re

N, Mi

N, Mo

N, Re

**Supplementary Figure S8: Random Forest analysis of VOCs from stage 2 olive oil (A)** Random Forest identifies the 15 most discriminatory VOCs across all samples; CAP models were produced for samples from different locations (Mirto Crosia (Mi), Mongrassano (Mo), Rende (Re)) using relative abundance of the three most discriminatory VOCs: hexanal (C40), methyl acetate (C26) and 3-hexen-1-ol (C20) according to **(B)** location **(C)** cultivars (‘Nocellara messinese (No), ‘Carolea’ (C)) and cultivars x location **(D).** The first two linear discriminants were used for the CAP analysis and each ellipse represents the 95% confidence interval (S.D.). Percentage of correct classification where n= 6, 9 and 3 respectively, was (B) 94%, (*P* = 0.69) (C) 100%, (P = 0.01) and (D) 89%, (P = 1).
